# Teacher-Student Interactions of Autistic Adolescents: Relationships between Teacher Autonomy Support, Structure, Involvement and Student Engagement

**DOI:** 10.1007/s10803-025-06723-0

**Published:** 2025-02-14

**Authors:** Fernanda Esqueda Villegas, Steffie van der Steen, Marijn van Dijk, David Alejandro Esqueda Villegas, Alexander Minnaert

**Affiliations:** 1 Department of Inclusive and Special Needs Education, Faculty of Behavioural and Social Sciences, University of Groningen, Grote Kruisstraat 2/1, Groningen, 9712 TS The Netherlands; 2 Department of Integral Postgraduate Studies in Social Sciences, Faculty of Interdisciplinary Studies in Social Sciences, University of Sonora, Blvd. Luis Encinas J, Av. Rosales and Centro, Hermosillo, Sonora 83000 Mexico

**Keywords:** Autism spectrum disorder, Self-determination theory, Student engagement, Classroom observations, State space grids, Inclusive education

## Abstract

The extent to which autistic students are provided with autonomy-support, structure and involvement during the teacher-student interaction has been under-researched. Few studies have focused on autistic secondary school students and even fewer have used observations to analyze their interactions. In this research, we aimed to understand the interaction dynamics between autistic students and their teachers in the Netherlands and Mexico; two countries committed to inclusive education. Six teacher-student dyads from five mainstream secondary schools in the Netherlands and seven teacher-student dyads from one school in Mexico participated. We used a fine-grained observational method to describe the classroom interactions, systematically coded from the lens of Self-Determination Theory. To study the interaction as a whole and co-occurring teacher and student behaviors (rather than breaking it down into separate parts), we used State Space Grids. Teacher-student interactions varied widely. In general, Dutch and Mexican teachers provided much structure in the classroom, which (in many cases) coincided with the active engagement of autistic students. At the same time, autistic students in both countries received little autonomy-support. However, when these students received autonomy-support, they responded with more engagement. In contrast, both engaged and disengaged student behaviors occurred when the teacher showed involvement. The desired patterns of interaction were not observed in all teacher-student pairs. Our findings indicate that teachers in both countries are missing opportunities to be autonomy-supportive. This contextual factor was minimally observed in the teacher-student interactions, particularly when compared to the levels of structure and involvement provided during lessons.

**Supplementary Information:**

The online version contains supplementary material available at 10.1007/s10803-025-06723-0.

## Introduction

Research has demonstrated that autistic students are less engaged with classroom tasks and in the school setting (Jahromi et al., 2013; Roorda et al., 2021; Zajic et al., 2020). One predictor of their engagement is the quality of the teacher-student relationship (Losh et al., 2022; Roorda et al., 2021). Unfortunately, autistic students in primary education have a high risk of developing poorer teacher-student relationships (Blacher et al., 2014; Caplan et al., 2016; Feldman et al., 2019; Zee et al., 2020). This is worrisome because these studies were conducted at the primary level, where the teacher-student bond is thought to be stronger than at the secondary level, which often has rotating rather than permanent teachers (Breault, 2013). Whether poorer teacher-student relationships are a recurring phenomenon for autistic students during their school years remains to be investigated, as few studies have focused on autistic adolescents in secondary education (Hill, 2014), and even fewer have used observations to analyze the teacher-student interaction as it occurs in real time (Esqueda Villegas et al., 2024). The present study therefore looked into the observable interaction dynamics of autistic adolescents and their teachers, using the theoretical framework of Self-Determination Theory (SDT).

SDT establishes that all individuals have three basic psychological needs for autonomy, competence, and relatedness (Ryan & Deci, 2000, 2018). Teachers can help to fulfill these needs in the classroom context by providing autonomy-support, structure, and showing involvement during their lessons and in the interactions with their students (Snikkers-Mommer et al., 2024). Autonomy-supportive teachers acknowledge students’ needs and interests (Reeve & Jang, 2006); they provide students with choices and implement classroom activities that are meaningful (Jang et al., 2010; Ryan & Deci, 2018). Teachers can foster structure by providing step-by-step guidance to their students during a lesson, communicating clearly what is expected from them, and giving constructive feedback (Jang et al., 2010; Skinner & Belmont, 1993). Additionally, teachers can offer structure when they present an overview of the lesson organization and build on previous lessons to introduce new knowledge (Maulana et al., 2012). To demonstrate involvement, teachers can show responsiveness by listening actively, showing support, and taking time to address students’ questions (Ryan & Deci, 2018). The assumption is that when the three basic psychological needs are met, students will feel engaged in their learning (Ryan & Deci, 2018). Notably, previous studies suggest that each component (of need-supportive teaching) contributes uniquely to students’ engagement (Jang et al., 2010; Loopers et al., 2024; Maulana et al., 2013; Snikkers-Mommer et al., 2024).

Some teaching methods or classroom strategies can be counterproductive and thwart students’ need for autonomy, competence, and relatedness. For example, teachers may thwart students’ autonomy by using controlling language, or by giving commands about what students “must” do during the lesson (Reeve & Jang, 2006). In addition, they may give unclear or vague instructions and be unavailable to answer students’ questions due to various reasons (Stroet, 2014), which could be considered as chaos. Lastly, teachers can display disaffection when they address students in an unfriendly tone or fail to show commitment and support (Ryan & Deci, 2018; Stroet, 2014). All of these teacher behaviors can have a negative impact on student engagement.

Despite the literature emphasizing the relevance of both SDT and the teacher-student interaction to foster students’ engagement, the extent to which autistic students are provided with autonomy-support, structure, and involvement during the teacher-student interaction has been under-researched. However, the three dimensions of SDT have been (indirectly) addressed by a few studies. In terms of autonomy, research indicates that teachers perceive (inclusive) education as a means through which autistic students can become more autonomous and independent (Cassimos et al., 2015). In a more recent study, Tesfaye et al. (2023) found that autistic youth (aged 11–18 years) valued making their own decisions, particularly if these concern them directly. Yet, it seems like schools rarely provide opportunities for autistic students to experience autonomy, although this might increase their motivation and engagement (Heyworth et al., 2021; Shea et al., 2013). Regarding structure, this type of need-support is highly appreciated by autistic students (Saggers et al., 2011). Without guidance and clear instructions on what they have to do, autistic students have reported to experience ‘mental breakdowns’ and feel ‘panicked’ or ‘stressed’ in secondary schools (Esqueda Villegas et al., submitted). In terms of involvement, autistic students value teachers who are kind, check up on them throughout the day, show understanding and are active listeners (Saggers et al., 2011; Sproston et al., 2017; Stephenson et al., 2021). Lastly, these students value teachers who recognize their needs and care about establishing a personal connection (Goodall, 2019; Humphrey & Lewis, 2008). In short, although most studies do not observe what happens during teacher-student interactions in the classroom, previous research does underscore autistic students’ need for a classroom context that provides autonomy support, structure, and involvement. To capture what occurs during real-time classroom interactions in terms of teachers’ need-support and student engagement, we propose the teacher-(autistic) student interaction as a complex dynamic system.

### The Teacher – (Autistic) Student Interaction: A Complex Dynamic System

A handful of researchers have examined real-time teacher- (non-autistic) student interactions in the classroom, inspired by the theory of complex dynamic systems (Loopers et al., 2023; Pennings & Mainhard, 2016; Smit et al., 2022; Zeinstra et al., 2023). Within this approach, behaviors are established as *states* that change from moment-to-moment in real-time interactions (Hollenstein, 2013). In this paper, a *state* represents a combination of a teacher’s (lack of) need-support (autonomy-support, structure, or involvement) and a student’s behavioral (dis)engagement. A *state space* encompasses all possible states that can occur, with some behavioral combinations being more likely to occur than others (Hollenstein, 2013; Pennings & Hollenstein, 2020). The most frequent interaction combinations, which the teacher-student dynamic is ‘pulled into’, are known as *attractor states*. Hollenstein (2013) recognizes two types of attractor states: shallow (weak) and deep (strong). In case of a shallow attractor state, it is easier for the teacher-student system to move from one state to another, thus they show more variability in the way they interact. Conversely, a *deep attractor state* is a teacher-student behavioral pattern less flexible, more rigid, and harder to break (Hollenstein, 2013). Notably, attractor states can be either desirable or undesirable interaction patterns (Hollenstein, 2013). For example, in a hypothetical ‘desirable’ attractor state, the teacher provides structure to the autistic student and the student shows engagement by interacting with the teacher/class, the task, or listening. In contrast, in an ‘undesirable’ attractor state, the teacher-student interaction is pulled into the dynamic of the teacher being controlling and the student being off-task. In time, behaviors tend to stabilize and become more difficult to change (Turner & Christensen, 2020), regardless of whether these are characterized by undesirable or desirable interactional patterns.

### Educational Context of the Netherlands and Mexico

The current study took place in two member states of the United Nations with two different approaches to secondary education: the Netherlands and Mexico. According to the OECD (2016), the Dutch education system ‘stands out from the crowd’ as there is no national curriculum to be followed and schools have the autonomy to decide *what* and *how* topics are being taught. In general, the Dutch secondary education system is divided in three levels of secondary education: pre-vocational secondary education [VMBO; four grades], senior general secondary education [HAVO; five grades], and pre-university education [VWO or Gymnasium; six grades]. Enrollment takes place after primary school around age 12 and admission is decided by secondary schools in cooperation with the student’s primary school (Ministry of Education, Culture and Science, 2007). The most recent State of Education report found that the majority of Dutch teachers usually give clear explanations of the teaching materials and provide students with a safe learning environment (Inspectorate of Education, 2023). However, it was also observed that many teachers do not explicitly state the aim of lessons or emphasize the relevance of learning specific topics, meaning there is room for improvement in their teaching practices (Inspectorate of Education, 2023). Lastly, some researchers have pointed out that non-inclusive practices, such as not providing differentiated instruction, still take place in Dutch schools (van Doodewaard & Knoppers, 2021).

Mexico is recognized as having the most diverse and complex educational system among the OECD countries (OECD, 2018, 2019a, b). In Mexico, secondary education is structured in two levels: lower secondary education (3 grades) and upper secondary education (general or vocational programs; 3 grades). The system is further divided into public and private education, with the latter offering advantages in terms of teaching quality and physical resources (Trevino, 2015). Student enrollment in lower secondary education takes place between 11 and 14 years old, while in upper secondary education is between ages 15 and 18 (Gobierno de México, 2022b). Notably, school attendance is mandatory until upper secondary education (Santiago et al., 2012). While several reports provide an overview of Mexico’s educational system, there is limited information about *how* children with special educational needs are being supported (García-Cedillo et al., 2015; Gobierno de México, 2022a). The few studies available suggest that Mexican teachers have a positive view on inclusive education (Francis et al., 2021; Lavin et al., 2022). However, teachers in secondary education feel less positive about their interactions with students with disabilities, while others have concerns about how inclusive their teaching practices are (Forlin et al., 2010). In sum, these two countries committed to inclusive education offer an interesting perspective to explore similarities and differences in the interaction between teachers and autistic students.

In the current study, we used State Space Grids to understand the interaction dynamics between teachers’ need support (autonomy-support, structure and involvement) and (autistic) students’ (dis)engagement during two consecutive lessons. The following research questions guided our exploratory study:

RQ1: How are teacher-student interactions characterized in terms of their dynamics in the teacher (autonomy, structure and involvement) and student (dis-engagement) dimensions?

RQ2: How are the teacher-student interactions characterized when teachers provide particularly high or low proportions of autonomy support, structure, or involvement?

RQ3: What are the similarities and differences in the most frequently observed teacher-student interaction patterns between Mexico and the Netherlands?

## Method

### Participants

Our sample comprised six teacher-student dyads from five schools in the Netherlands and seven teacher-student dyads from one school in Mexico. Teachers from the Netherlands taught Math, English, or Dutch. Dutch teachers hold an (applied) university Bachelor degree to teach in the lower secondary education levels (VMBO; pre-vocational secondary education) and a University degree to teach the higher levels (HAVO; senior general secondary education and VWO; pre-university). Teachers from Mexico taught Physics, Math, Sociology, or English. Mexican teachers hold a Bachelor degree and a qualification in ‘Skills for teaching in secondary education’. In addition, English teachers have a National Certification of the English Language (CENNI, in Spanish) of level ‘Intermediate Superior’. Regarding the students, all autistic participants had a formal Autism Spectrum Disorder (ASD) diagnosis according to the DSM-V. Furthermore, they had IQs within the average range (typically between 90 and 110), and they demonstrated both receptive and expressive language skills. Notably, these data were discussed with the school coordinators and no additional measurement instrument was applied. The male-to-female ratio of autistic Dutch students was 4:2, with an average age of 15 years at the time of data collection. The male-to-female ratio of autistic Mexican students was 6:1, with a mean age of 16.5 years. Sociodemographic characteristics of Dutch and Mexican participants are provided in Tables 1 and 2, respectively.

**Table 1:** Sociodemographic information of Dutch participants

School pseudonym	School level	Teacher pseudonym	Subject	Duration of the lesson	School grade	Student pseudonym	Gender	Age at the time of data collection
Secondary School 1	HAVO	Richard	Math	L1: 43 min L2: 90 min	4th	David	Male	15 years old
						Alan	Male	15 years old
Secondary School 2	VMBO	Maria	English	L1: 40 min L2: 31 min	3rd	Rachel	Female	15 years old
Secondary School 3	VMBO	Anne	Dutch	L1: 45 min L2: 45 min	1st	Alex	Male	13 years old
Secondary School 4	VWO	Jack	Math	L1: 41 min L2: 41 min	4th	Sandra	Female	16 years old
Secondary School 5	HAVO	Henry	Math	L1: 45 min L2: 48 min	4th	Simon	Male	16 years old

*Note. VMBO =* Pre-vocational secondary education, HAVO = Senior general secondary education, and VWO = Pre-university education. L1 refers to Lesson 1 and L2 refers to Lesson 2

**Table 2:** Sociodemographic information of Mexican participants

School pseudonym	Teacher pseudonym	Subject	Duration of the lesson	School grade	Student pseudonym	Gender	Age at the time of data collection
Secondary School A (Public school)	Rafael	Physics	L1: 46 min L2: 41 min	2nd	Sebastian	Male	17 years old
			L1: 40 min L2: 31 min	2nd	Cesar	Male	17 years old
	Erick	Math	L1: 43 min L2: 40 min	2nd	Adrian	Male	16 years old
			L1: 40 min L2: 41 min	2nd	Daniel	Male	17 years old
	Cindy	Sociology	L1: 31 min L2: 40 min	3rd	Alberto	Male	unknown
					Jesus	Male	17 years old
	Sofia	English	L1: 41 min L2: 44 min	1st	Sara	Female	15 years old

*Note.* Officially, lessons last 50 min. However, in some cases the teacher arrived later and therefore lessons were shorter. L1 refers to Lesson 1 and L2 refers to Lesson 2

### Procedure

Mainstream secondary schools were contacted by graduate students in the Netherlands and by the first author in Mexico. In subsequent meetings, school principals and/or coordinators were informed about the study. Information sheets explaining the goal of the study and the procedure were shared with teachers, parents, and autistic students. Additionally, the parents of the other students were informed about this study. However, in order to avoid flagging the autistic student, the other parents were told that the project focused on classroom interactions in general. Parents could object to their child being recorded on the camera and these students would switch seating places. Additionally, all the faces and bodies of non-participants were blurred by the first author after recording and excluded from data analysis. Active informed consent was obtained from teachers, parents and autistic students. For Mexican participants, an additional consent form was signed by the school principal. Participation was completely voluntary and no monetary compensation was offered. Ethical approval was granted by the Pedagogy and Educational Sciences ethics review chamber from the Faculty of Behavioural and Social Sciences at the host university.

After written consent was obtained, the scheduling of the recording was discussed with the school coordinator or teachers. Two lessons for each teacher-student dyad were recorded using two cameras: one aimed at the teacher and one at the entire class. Teachers were instructed to implement their lessons as usual. On average, the duration of lessons in the Netherlands was 46.9 min, while lessons in the Mexican setting had an average duration of 39.8 min. Data were pseudonymized and securely stored in the (online) environment of the host university.

### Measurements – Coding Scheme

We coded teachers’ utterances that targeted the entire class, the autistic student, or a group of students to which the autistic student belonged, thereby omitting more individual interactions between the teacher and other (specific) students. Building on previous SDT classroom research (Loopers, 2022; Stroet, 2014; Stroet et al., 2013), we constructed a coding scheme to code teachers’ utterances for each SDT dimension separately: autonomy support/thwart, structure/chaos, and involvement/disaffection. Autonomy-support was defined as teacher behaviors that encouraged student choice and acknowledged students’ needs and interests (Jang et al., 2010; Reeve & Jang, 2006). In contrast, autonomy thwart included instances of controlling language or pressuring students to complete tasks (Reeve & Jang, 2006). Structure encompassed teacher actions that provided clear expectations, guidance, and constructive feedback (Jang et al., 2010; Skinner & Belmont, 1993), while chaos described situations where the teacher’s instructions were unclear (Stroet, 2014). Involvement was defined as behaviors reflecting teacher’s supportiveness and attention to students’ questions (Ryan & Deci, 2018). Conversely, disaffection included instances of unfriendliness or lack of commitment to students’ learning (Ryan & Deci, 2018; Stroet, 2014). All dimensions had a neutral category for when no specific need support or lack of thereof was observed.

We conceptualized the three dimensions as independent from one another, since a *single* teacher utterance can be, for instance, both reflecting autonomy support *and* structure (Jang et al., 2010). For example, the utterance marked as ‘Autonomy thwart’[Alright, youngsters. I want to see you working. On your task. I don’t want to see you slacking off] in the ‘Autonomy support/thwart’ dimension (see Table 3), was coded as ‘Neutral’ in the ‘Structure/Chaos’ dimension and coded as ‘Disaffection’ in the ‘Involvement’ dimension. This means that each single teacher utterance was coded on all three dimensions. Table 3 provides a shortened version of the coding scheme of teachers’ behaviors with examples.

To code the student behaviors, we developed a coding scheme based on previous research on student engagement (Christenson et al., 2012). Engaged states (and therefore ‘desirable’ student states) included interacting with the teacher/class, interacting with a task, and listening/paying attention. Disengaged states (and therefore ‘undesirable’ student states) encompassed being off-task active (i.e., disrupting classmates) or off-task passive (i.e., staring out the window). The state ‘Other’ was used for instances such as waiting in line, briefly leaving the classroom (i.e., to use the restroom), or if the student behaviors did not fall into any other state. Table 4 provides a simplified version of the coding scheme for students’ behaviors. The complete coding protocol can be found in the Supplementary Materials.

**Table 3:** Coding scheme for teacher’s behaviors, including examples

Dimension	Codes	Description	Examples
1. Autonomy support/ thwart	Autonomy-support	The teacher allows the student to make decisions. The ideas and suggestions from the student are appreciated. The teacher lets the student(s) know the importance of the tasks they will perform.	*You can choose which paragraph you want to work on first.* *It is online [the exam]. Try to do it tomorrow so that I can help you if there is a technical problem.*
	Neutral	If an utterance does not fall under the category autonomy-support or thwart, it should be coded as ‘Neutral’.	*In English*,* we use 2 words to talk about “the future”.*
	Autonomy thwart	The teacher uses controlling language. The teacher sets strict rules or the pace in which students have to work. The teacher does not explain why a task is important or relevant and forces the student to do the activity.	*Alright*,* youngsters. I want to see you working. On your task. I don’t want to see you slacking off.* *Okay*,* if you wrap it up*,* please. Three… two… one…*
2. Structure/ Chaos	Structure	The teacher is explicit with regard to what the students have to learn, what they have to do and how. The teacher provides step-by-step directions on what the student has to do during an on-going activity. The teacher discusses what the students learned or did during the lesson and provides instructions for what follows next (such as homework).	*Today*,* we are going to start with Question 1 on page 6 and we are going to learn how to…* *Last class we were working with the properties of logarithms[…]. Don’t forget to do the assignments and remember that the exam is tomorrow.*
	Neutral	If an utterance does not fall under the category structure or chaos, it should be coded as ‘Neutral’.	*X* ^2^ *divided by X.*
	Chaos	The instructions provided by the teacher are confusing and unclear. The teacher uses verbal behavior to punish the student. The teacher provides negative or purely evaluative feedback when the student does not have the right answer.	*You can do part A or part B*,* maybe we get to the explanation of part C*,* but we have to see*,* maybe not.* *It’s the one here; in the middle*,* okay? Okay*,* not exactly in the middle. More like over there.*
3. Involvement/ Disaffection	Involvement	The teacher addresses the students with a friendly tone; shows concern. The teacher is approachable and available for the students. The teacher is caring and supportive towards the student(s).	*Do not be worried about the exam. It will cover topics that we’ve already looked into.* *Does anyone have a question about the exam or previous classes?*
	Neutral	If an utterance does not fall under the category involvement or disaffection, it should be coded as ‘Neutral’.	*Take out your books. Page 128.*
	Disaffection	The teacher talks to the student in an unfriendly tone and treats him/her unfriendly. The teacher is not available when the student looks up for him/her.	*Continue reading. Keep reading. Keep reading* [in a pressuring tone]. *Finish reading it* [the task] *and then you’ll find out* [the answer] *No. We do not have time for questions.*

**Table 4:** Coding scheme for student’s behaviors, including descriptions

Category	Description
Interacting with the teacher and/or the class	The student is actively involved and interacting with the teacher by contributing to the lesson. The student shares ideas or findings. Signs: *Raising hand*,* contact with the teacher*,* responsive to teacher’s question*,* little distraction.*
Actively interacting with a task	The student is actively involved in a given task, such as doing an activity, working on a problem or reading, and seems to be concentrated and interested in it. This behavior can also occur while the teacher is giving instructions. The student does not verbalize anything here. Signs: *Not easily distracted by others*,* eye-gazed focused on the whiteboard or book*,* taking notes.*
Listening and paying attention	While the teacher is explaining the lesson or activity, the student is listening and paying attention. Signs: *Eye-gazed focused on the teacher or the student who is speaking.*
Off-task active	The student initiates the off-task behavior by disrupting a classmate or interrupting the teacher with a non-academic question/comment. The student can also be manipulating objects that are not needed for the task. Signs: *Joins off-topic conversations*,* interruptions*,* playing with materials that are not used during the activity.*
Off-task passive	The student is not at all – or barely – focused on the given task. The student appears to be daydreaming, distracted or is listening to a classmate’s off-task contribution. Signs: *procrastination*,* sighing*,* easily distracted*,* staring at the window/door*,* gaze at other students who are not working on the task.*
Other	The student is standing in line waiting for the teacher to revise the task; the student leaves the classroom to use the restroom.

### Coding Procedure

All coders received a three-hour training in which SDT, the protocol and coding procedure were explained. Additionally, examples of teacher utterances and segments from a lesson (in which student behaviors could be observed) were provided. The online program Mediacoder was used to code the lessons (Bos & Steenbeek, 2017). To code the teachers’ utterances, we used the camera focused on the teacher. For the students’ behaviors, the camera focused on the entire class was used. Two independent researchers coded 15% of each lesson (12 Dutch lessons or 14 Mexican lessons). If the percentage of inter-rater reliability was at least 80%, the remainder of the lesson could be coded by one of the researchers. In cases where agreement fell below this level, discrepancies were discussed and an additional 15% of the lesson was coded independently until the desired level of interrater agreement was reached. Table 5 provides the final average agreement percentages (based on all videos) for each dimension.

**Table 5:** Average percentages of agreement for Dutch and Mexican participants

	Dimension			
	Autonomy/Control	Structure/Unclear	Relatedness/Disaffection	Students’ behaviors
Dutch participants	85%	91%	94%	91%
Mexican participants	92%	87%	94%	93%

### State Space Grids Data Analyses

Once all lessons were coded, we coupled the teacher’s codes and student’s codes for each SDT dimension: autonomy support/thwart, structure/chaos, involvement/disaffection. For convenience purposes, we refer to teachers’ autonomy-support, structure, and involvement as ‘desirable’ teacher states, whereas teachers’ autonomy thwart, chaos, and disaffection are considered ‘undesirable’ teacher states. Engaged students’ states (interacting with the teacher/class, interacting with a task, and listening) are regarded as ‘desirable’ student states, while disengaged states (off-task passive and off-task active) are seen as ‘undesirable’ student states. Using the software Gridware, we constructed State Space Grids for each teacher-student dyad (Hollenstein, 2013; Lamey et al., 2004). This method allowed us to visualize the intersection between two-dimensional state spaces (teacher and student states) and obtained several dynamic interaction measures (Hollenstein, 2013; Lamey et al., 2004). That being said, an event in the grid is one teacher action which is immediately followed by a student action. This is illustrated as a dot in the grid. State Space Grids for each teacher-student pair can be found in the Supplementary Materials.

**Fig. 1:**
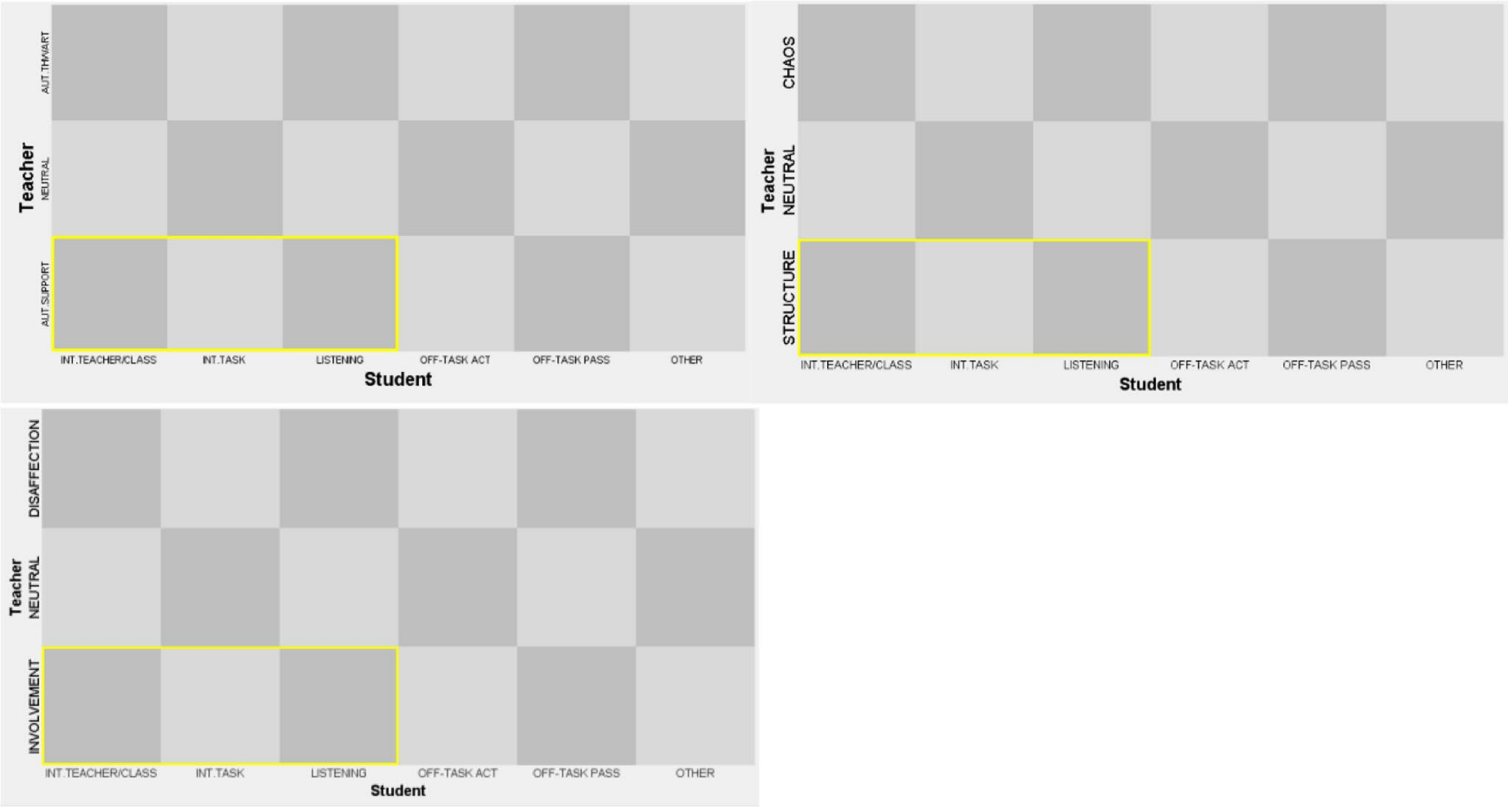
Desirable zones for the autonomy-support, structure, and involvement dimensions

To address RQ1 (how the teacher-student interactions are characterized in terms of their dynamics), we analyzed the number of visits to determine the attractor states in each lesson, which underline the patterns that are more likely to occur than others. Additionally, we calculated each dyad’s percentage in the ‘desirable’ zone (see Fig. 1) of each dimension by dividing the number of ‘desirable cell visits’ by the total number of ‘events’ within the full grid. Lastly, we computed dispersion values of each teacher-student dyad. Dispersion values are calculated with the formula $\:1-\frac{\left(n\:\sum\:\:\left({d}_{i/D}\right)2\right)-1}{n-1}$ and range from 0 to 1; where 0 means that *all* interactions are concentrated in a single cell, and 1 indicates that interactions are spread across *all* grid cells (Hollenstein, 2013). Therefore, lower dispersion values indicate fairly rigid teacher-student interactions, while higher dispersion values suggest (more) flexibility.

For RQ2, we identified the dyads with particularly high or low proportions of (teacher) autonomy-support, structure and involvement to focus on. Subsequently, we selected regions of interest (i.e., the teacher desirable states ‘autonomy’, ‘structure’, or ‘involvement’ and *all* the possible student states) for these dyads during their two lessons and discussed the main characteristics of these interactions, such as the visited states and attractor states. Lastly, for RQ3 (similarities and differences between countries), we performed group-based analyses combining *all* lessons within each country and described the most frequent interaction patterns within each country (using the ‘number of visits’ measure) when teachers provided autonomy-support, structure and involvement.

## Results

### Interaction Dynamics in the (Teacher) Autonomy-Support and (Student) Engagement Dimension

The ‘desirable zone’ in which teachers provide autonomy-support and students are engaged by interacting with the teacher/class, a task or listening, was visited on average 2.6% of the time (range: 0–7%) across lessons and countries. Therefore, none of the dyads in our sample had a teacher whose most frequent pattern was providing autonomy-support. This means that students were *rarely* provided with choices. Instead, teachers hardly moved from being ‘Neutral’, while the students moved in-and-out across the different states, such as ‘Listening’, ‘Interacting with the task/teacher/class’, or ‘Off-task passive’. Mutual variation was therefore not observed. Yet, most of the dispersion values oscillated between 0.7 and 0.9 (recall that values can range from 0—very rigid, to 1—very flexible) due to the variation in student behaviors, moving from one state to another. An exception to this was a teacher-student pair who seemed to be “stuck” in the student being ‘Off-task passive’ while the teacher was ‘Neutral’ (dispersion value of 0.38). Other teacher-student dyads with a disengaged student state (‘Off-task passive’) showed more variability in their interaction (see Table 6).

**Table 6:** Characteristics of the teacher-student interactions in the teacher ‘autonomy-support’ and student engagement dimensions

Teacher – Student dyad	Cell range	Attractor Teacher – Student	Dispersion	% in the desirable zone
*Dutch participants*				
Richard – David 1	6	Neutral/Listening	0.593	0.9
Richard – David 2	6	Neutral/Listening	0.755	0.1
Richard – Alan 1	7	Neutral/Listening	0.718	0.9
Richard – Alan 2	7	Neutral/Interacting with a task *and* Neutral/Listening	0.779	0.1
Maria – Rachel 1	7	Neutral/Listening	0.822	0.0
Maria – Rachel 2	5	Neutral/Listening	0.723	0.0
Anne – Alex 1	12	Neutral/Off-task passive	0.881	1.1
Anne – Alex 2	14	Neutral/Interacting with a task	0.904	6.3
Jack – Sandra 1	7	Neutral/Interacting with a task *and* Neutral/Listening	0.751	6.1
Jack – Sandra 2	8	Neutral/Interacting with a task	0.823	6.7
Henry – Simon 1	10	Neutral/Off-task passive	0.865	0.6
Henry – Simon 2	8	Neutral/Off-task passive	0.382	2.4
*Mexican participants*				
Rafael – Sebastian 1	10	Neutral/Listening	0.811	2.3
Rafael – Sebastian 2	9	Neutral/Listening	0.81	1.4
Rafael – Cesar 1	10	Neutral/Interacting with teacher/class *and* Neutral/Interacting with a task	0.784	3.2
Rafael – Cesar 2	11	Neutral/Listening	0.815	2.3
Erick – Adrian 1	11	Neutral/Listening	0.82	7.0
Erick – Adrian 2	13	Neutral/Listening	0.896	2.0
Erick – Daniel 1	11	Neutral/Listening	0.721	4.9
Erick – Daniel 2	10	Neutral/Interacting with a task	0.804	2.1
Cindy – Alberto 1	6	Neutral/Listening *and* Neutral/Off-task passive	0.606	3.7
Cindy – Alberto 2	12	Autonomy thwart/Off-task passive *and* Neutral/Listening	0.913	4.5
Cindy – Jesus 1	7	Neutral/Off-task passive	0.761	6.1
Cindy – Jesus 2	14	Neutral/Off-task passive	0.923	3.0
Sofia – Sara 1	12	Neutral/Listening	0.864	3.7
Sofia – Sara 2	8	Neutral/Listening	0.479	4.7

Note The number “1” in the first column corresponds to Lesson 1 and “2” refers to Lesson 2. The column “Cell range” indicates the number of cells visited across the grid for this dimension, in which the maximum value is 18. Hence, a number closer to zero indicates more rigidness and a number closer to 18 more flexibility in the interaction. Dispersion is the underlying construct of flexibility/rigidity of the lesson. A value of ‘0’ would indicate that all behaviors are concentrated in one cell (and therefore, the behavior is rigid). A value of ‘1’ would be the maximum dispersion (and therefore, maximum flexibility) implicating that the behaviors are spread out evenly across the grid. The last column refers to the percentage that each teacher-student pair spent in the desirable ‘autonomy’ teacher state and the ‘engaged’ student states

### The Teacher-Student Interaction during Particularly High Proportions of Autonomy-Support

**Fig. 2:**
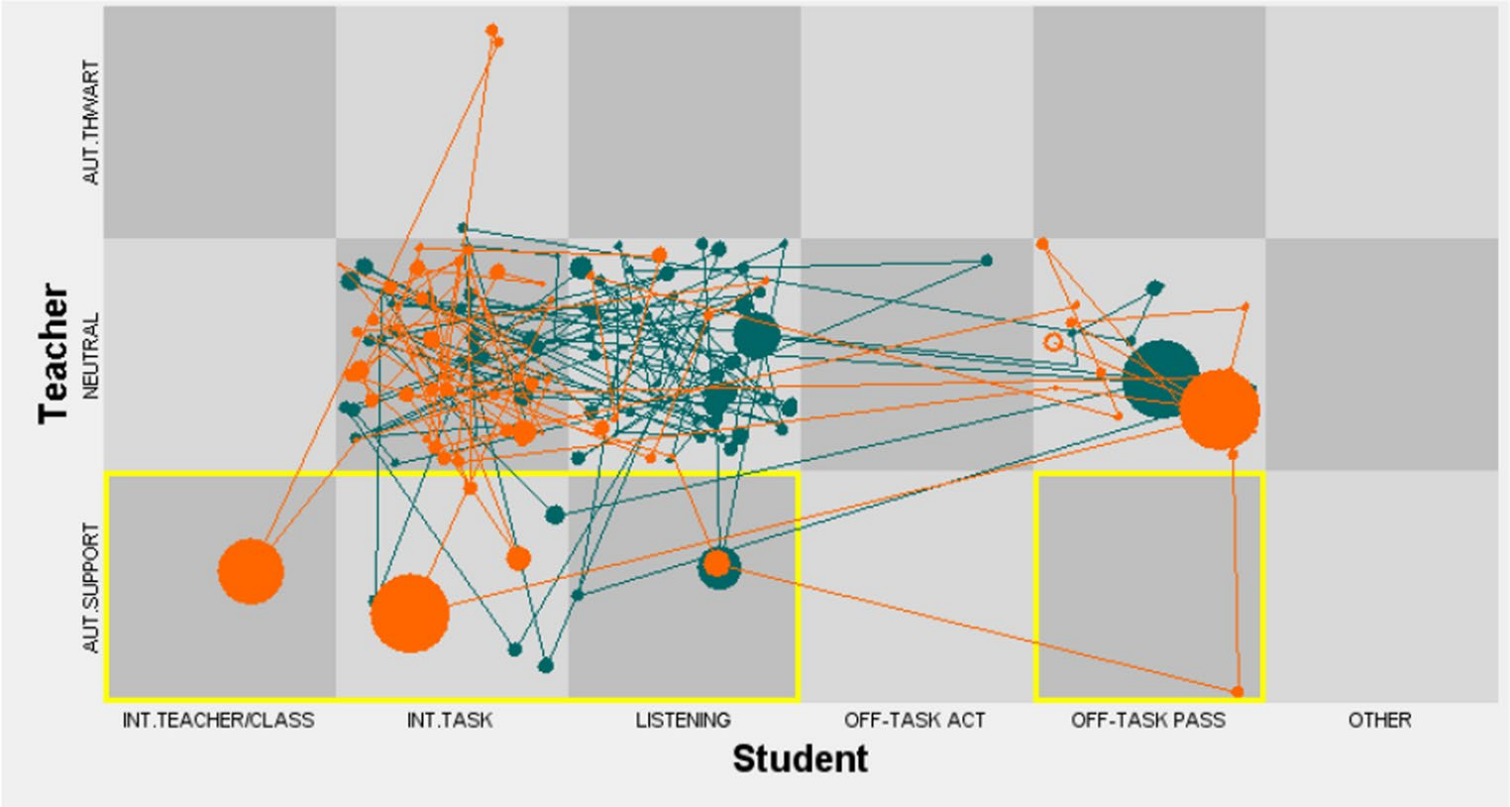
State Space Grid of lessons of Jack and Sandra (Dutch). The vertical axis illustrates the teacher states autonomy-support, neutral, and autonomy thwart. The horizontal axis shows all student states: ‘Engaged’ (interacting with teacher/class, interacting with a task, listening), ‘disengaged’ (off-task active, passive) and ‘other’. The region of interest is marked in yellow

‘Jack’, a Math teacher in the Netherlands, exhibited a relatively high proportion of autonomy-support across two lessons. This seemed to co-occur with more engagement of ‘Sandra’ (the student), as all engaged student states were visited. However, the attractor state (i.e., the most frequent pattern observed) was ‘Jack’ being ‘Neutral’ while Sandra was ‘Interacting with a task’ (see Fig. 2).

### The Teacher-Student Interaction During Particularly Low Proportions of Autonomy-Support

**Fig. 3:**
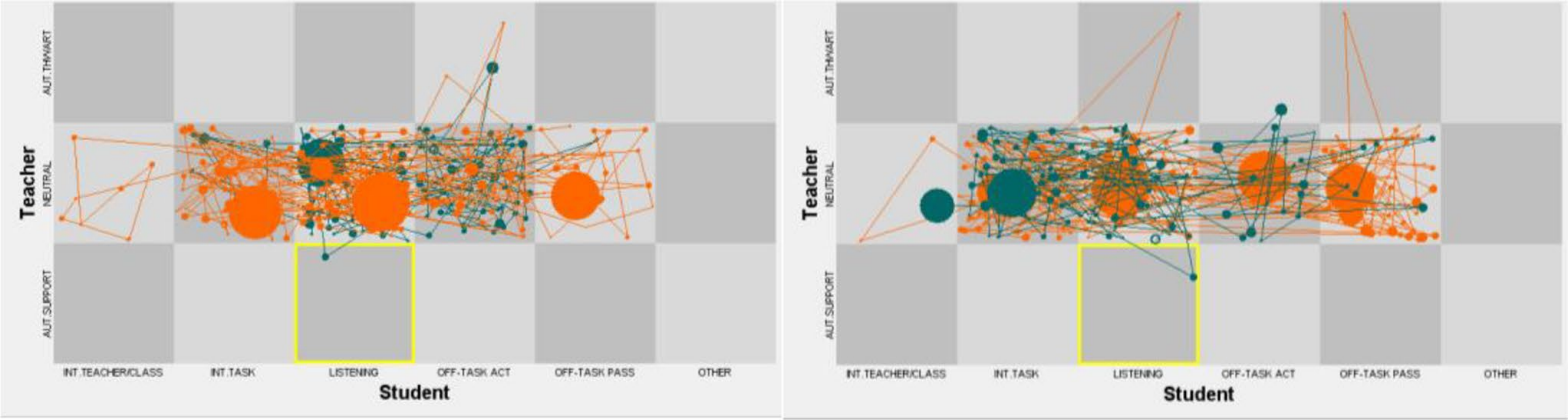
State Space Grids of lessons from Richard and David (left) and Alan (right)

‘Richard’, a Math teacher from the Netherlands, showed a relatively low proportion of autonomy-support. The most frequent interaction pattern observed was ‘Richard’ being ‘Neutral’ while students David (left figure) and Alan (right figure) were ‘Listening’. Notably, this teacher barely moved from the ‘Neutral’ state, while the students visited all engaged and disengaged states, making these interactions highly flexible (see Fig. 3).

When contrasting the two dyads with high and low proportions of autonomy-support, we can observe that more desirable student states co-occur when teachers provide (autistic) students with more opportunities to make their own choices.

### Interaction Dynamics in the (Teacher) Structure and (Student) Engagement Dimension

The ‘desirable zone’ in which teachers provide structure and students are engaged by either interacting with the teacher/class, a task or listening, was visited on average 15.0% of the time (range: 4.9–22.2%) across lessons and countries. Consequently, the majority of the interactions were characterized by teachers providing ‘Structure’. This means that students were often given step-by-step guidance, obtained feedback or were instructed on what they had to do. In the student states, we could observe that many of the autistic students were ‘Listening’ or ‘Interacting with a task’. However, in some lessons (seven), a commonly observed interaction pattern was the teacher giving ‘Structure’ and the student being either ‘Off-task active’ or ‘Off-task passive’, which seems counterintuitive. That said, most of the dispersion values fluctuated between 0.6 and 0.9 (range: 0—very rigid, to 1—very flexible), indicating that the teacher-student pairs could easily fluctuate between states (see Table 7).

**Table 7:** Characteristics of the teacher-student interactions in the teacher ‘structure’ and student engagement dimensions

Teacher – Student dyad	Cell range	Attractor Teacher – Student	Dispersion	% in the desirable zone
*Dutch participants*				
Richard – David 1	9	Neutral/Off-task active	0.791	9.2
Richard – David 2	11	Neutral/Listening	0.88	14.1
Richard – Alan 1	11	Neutral/Listening	0.892	15.6
Richard – Alan 2	10	Neutral/Interacting with a task	0.805	12.1
Maria – Rachel 1	11	Structure/Off-task active	0.919	17.6
Maria – Rachel 2	10	Neutral/Listening	0.755	16.4
Anne – Alex 1	15	Structure/Off-task passive	0.931	21.1
Anne – Alex 2	12	Structure/Interacting with a task	0.967	20.4
Jack – Sandra 1	9	Structure/Interacting with a task *and* Structure/Listening	0.698	22.2
Jack – Sandra 2	7	Structure/Interacting with a task	0.762	22.2
Henry – Simon 1	10	Neutral/Off-task passive *and* Structure/Off-task passive	0.821	14.6
Henry – Simon 2	8	Structure/Off-task passive	0.69	4.9
*Mexican participants*				
Rafael – Sebastian 1	11	Structure/Listening	0.727	14.9
Rafael – Sebastian 2	9	Structure/Listening	0.657	15.9
Rafael – Cesar 1	9	Structure/Interacting with a task	0.55	9.5
Rafael – Cesar 2	13	Structure/Listening	0.881	14.4
Erick – Adrian 1	8	Structure/Listening	0.73	9.6
Erick – Adrian 2	13	Structure/Listening	0.843	17.0
Erick – Daniel 1	11	Structure/Interacting with a task	0.691	17.1
Erick – Daniel 2	10	Structure/Interacting with a task *and* Structure/Off-task passive	0.786	11.0
Cindy – Alberto 1	7	Structure/Listening	0.797	17.1
Cindy – Alberto 2	15	Structure/Off-task passive	0.856	10.6
Cindy – Jesus 1	9	Neutral/Listening *and* Neutral/Off-task passive	0.888	17.1
Cindy – Jesus 2	13	Structure/Off-task passive	0.888	13.6
Sofia – Sara 1	12	Structure/Listening	0.823	16.7
Sofia – Sara 2	9	Structure/Listening	0.618	15.6

### The Teacher-Student Interaction During Particularly High Proportions of Structure

**Fig. 4:**
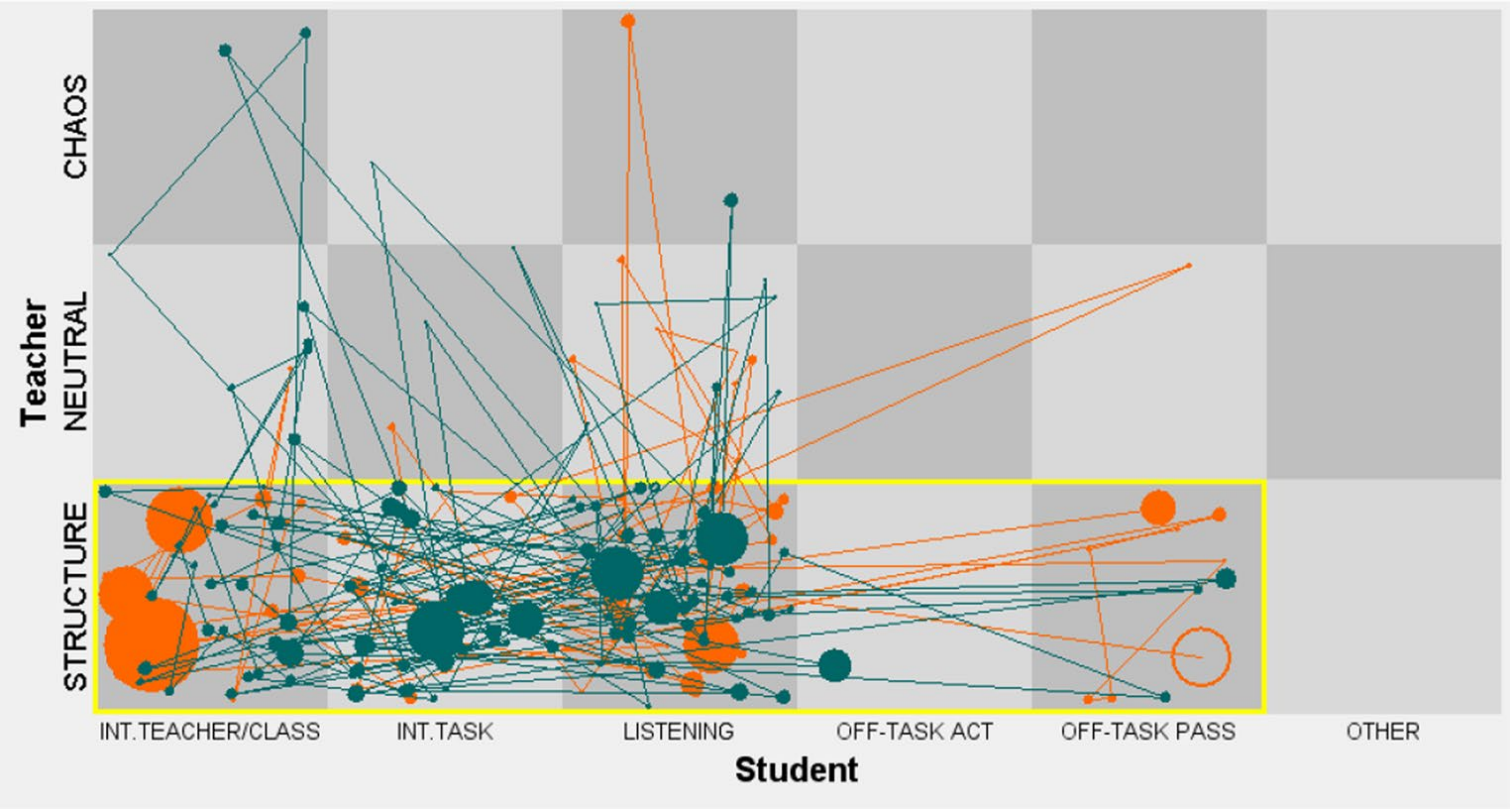
State Space Grid of the two lessons of Rafael and Sebastian. The vertical axis illustrates the teacher states structure, neutral, and chaos. The horizontal axis shows all student states: ‘Engaged’ (interacting with teacher/class, interacting with a task, listening), ‘disengaged’ (off-task active, passive) and ‘other’. The region of interest is marked in yellow

A Physics teacher in Mexico, ‘Rafael’, offered a high proportion of structure across two lessons. This co-occurred with both engaged and disengaged student states of ‘Sebastian’ (the student). However, the engaged states were visited more often than the disengaged ones, even when there was ‘Chaos’ on the teacher’s end. The most frequently observed interaction pattern was ‘Rafael’ providing ‘Structure’ while ‘Sebastian’ was ‘Listening’. To summarize, it seems that this highly structured lesson enhanced all types of engaged states for this autistic student (see Fig. 4).

### The Teacher-Student Interaction During Particularly Low Proportions of Structure

**Fig. 5:**
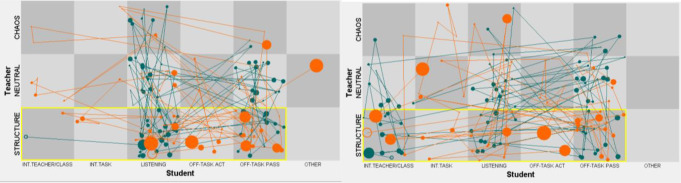
State Space Grids of lessons from Cindy and Alberto (left) and Jesus (right)

‘Cindy’, a Sociology teacher in Mexico, exhibited a low proportion of structure across our sample. In contrast with the previous teacher (Rafael), ‘Cindy’ moved much more across states, including ‘Chaos’. This co-occurred with less engagement of students ‘Alberto’ and ‘Jesus’ (See Fig. 5; left and right grids, respectively), as the disengaged student states were visited almost as often as the engaged ones. The most common interaction pattern observed was ‘Cindy’ providing ‘Structure’ while ‘Alberto’ was ‘Listening’ and ‘Jesus’ was ‘Off-task passive’. To summarize, less structure provided by this teacher seemed to co-occur with more disengaged student states.

When contrasting the two dyads with high and low proportions of structure, we can observe that the highly structured lesson co-occurred with more student engagement, even during (occasional) moments when the teacher was lacking clarity. Conversely, in the lesson that had less step-by-step guidance, more disengaged student states were observed.

### Interaction Dynamics in the (Teacher) Involvement and (Student) Engagement Dimension

The ‘desirable zone’ in which teachers show involvement and students are engaged by either interacting with the teacher/class, a task or listening, was visited on average 8.3% of the time (range 2.8–16.2%) across lessons. There was no attractor state in which the teacher was displaying involvement. A ‘Neutral’ teacher state was predominant while the student was ‘Listening’ or ‘Interacting with a task’. Notably, two teacher-student pairs were stuck in a disengaged student state (‘Off-task passive’) while the teacher was ‘Neutral’. This is particularly concerning for dyad Henry and Simon during lesson 2, as this pattern seems rather stable and harder to break, given that their dispersion value is 0.42. For most other pairs, the teacher-student interaction was highly variable in the involvement dimension (see Table 8), with most of the dispersion values ranging from 0.6 to 0.9 (values can range from 0—very rigid, to 1—very flexible interaction).

**Table 8:** Characteristics of the teacher-student interactions in the teacher ‘involvement’ and student engagement dimensions

Teacher – Student dyad	Cell range	Attractor Teacher – Student	Dispersion	% in the desirable zone
*Dutch participants*				
Richard – David 1	10	Neutral/Off-task active	0.674	8.3
Richard – David 2	12	Neutral/Listening	0.836	6.6
Richard – Alan 1	10	Neutral/Interacting with a task *and* Neutral/Listening	0.726	13.8
Richard – Alan 2	11	Neutral/Interacting with a task *and* Neutral/Listening	0.852	6.6
Maria – Rachel 1	14	Neutral/Listening *and* Neutral/Off-task active	0.961	9.9
Maria – Rachel 2	12	Neutral/Listening	0.853	14.7
Anne – Alex 1	15	Neutral/Interacting with a task *and* Neutral/Off-task passive	0.874	6.4
Anne – Alex 2	13	Neutral/Interacting with a task	0.927	9.8
Jack – Sandra 1	7	Neutral/Listening	0.827	15.2
Jack – Sandra 2	7	Neutral/Interacting with a task	0.836	16.2
Henry – Simon 1	10	Neutral/Off-task passive	0.796	4.5
Henry – Simon 2	9	Neutral/Off-task active *and* Neutral/Off-task passive	0.424	2.7
*Mexican participants*				
Rafael – Sebastian 1	11	Neutral/Listening	0.828	3.7
Rafael – Sebastian 2	9	Neutral/Listening	0.809	7.2
Rafael – Cesar 1	10	Neutral/Interacting with teacher/class	0.807	6.3
Rafael – Cesar 2	12	Neutral/Listening	0.811	5.3
Erick – Adrian 1	11	Neutral/Interacting with a task	0.809	12.2
Erick – Adrian 2	13	Neutral/Listening	0.915	11.0
Erick – Daniel 1	13	Neutral/Listening	0.831	12.2
Erick – Daniel 2	10	Neutral/Interacting with a task	0.791	2.8
Cindy – Alberto 1	6	Neutral/Listening	0.624	3.7
Cindy – Alberto 2	13	Neutral/Listening	0.931	9.1
Cindy – Jesus 1	7	Neutral/Off-task passive	0.763	4.9
Cindy – Jesus 2	14	Neutral/Off-task passive	0.928	10.6
Sofia – Sara 1	12	Neutral/Listening	0.854	11.1
Sofia – Sara 2	8	Neutral/Listening	0.516	9.4

### The Teacher-Student Interaction During Particularly High Proportions of Involvement

**Fig. 6:**
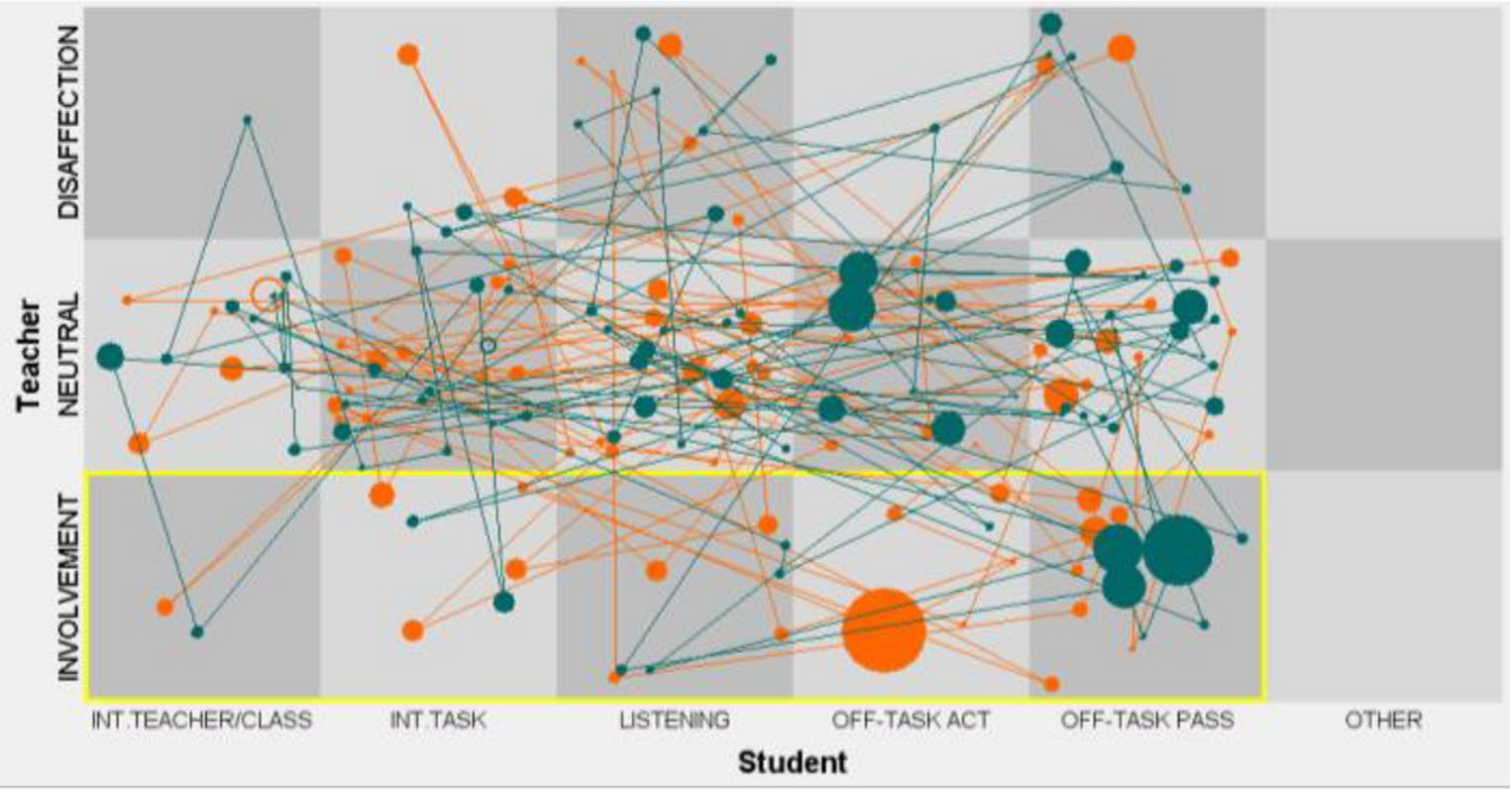
State Space Grids of the two lessons of Anne and Alex. The vertical axis illustrates the teacher states involvement, neutral, and disaffection. The horizontal axis shows all student states: ‘Engaged’ (interacting with teacher/class, interacting with a task, listening), ‘disengaged’ (off-task active, passive) and ‘other’. The region of interest is marked in yellow

‘Anne’, a Dutch teacher, showed a high proportion of involvement across two lessons. However, this did not seem to co-occur with more engagement of ‘Alex’ (the student), as engaged student states were visited as often as disengaged ones. Interestingly, teacher ‘Anne’ also showed disaffection at times, while the most frequent interaction pattern observed was ‘Anne’ being ‘Neutral’ while ‘Alex’ was ‘Interacting with a task’. In sum, this teacher-student interaction seemed highly flexible (with many different states visited), regardless of whether the teacher was caring, neutral or dismissive (see Fig. 6).

### The Teacher-Student Interaction During Particularly Low Proportions of Involvement

**Fig. 7:**
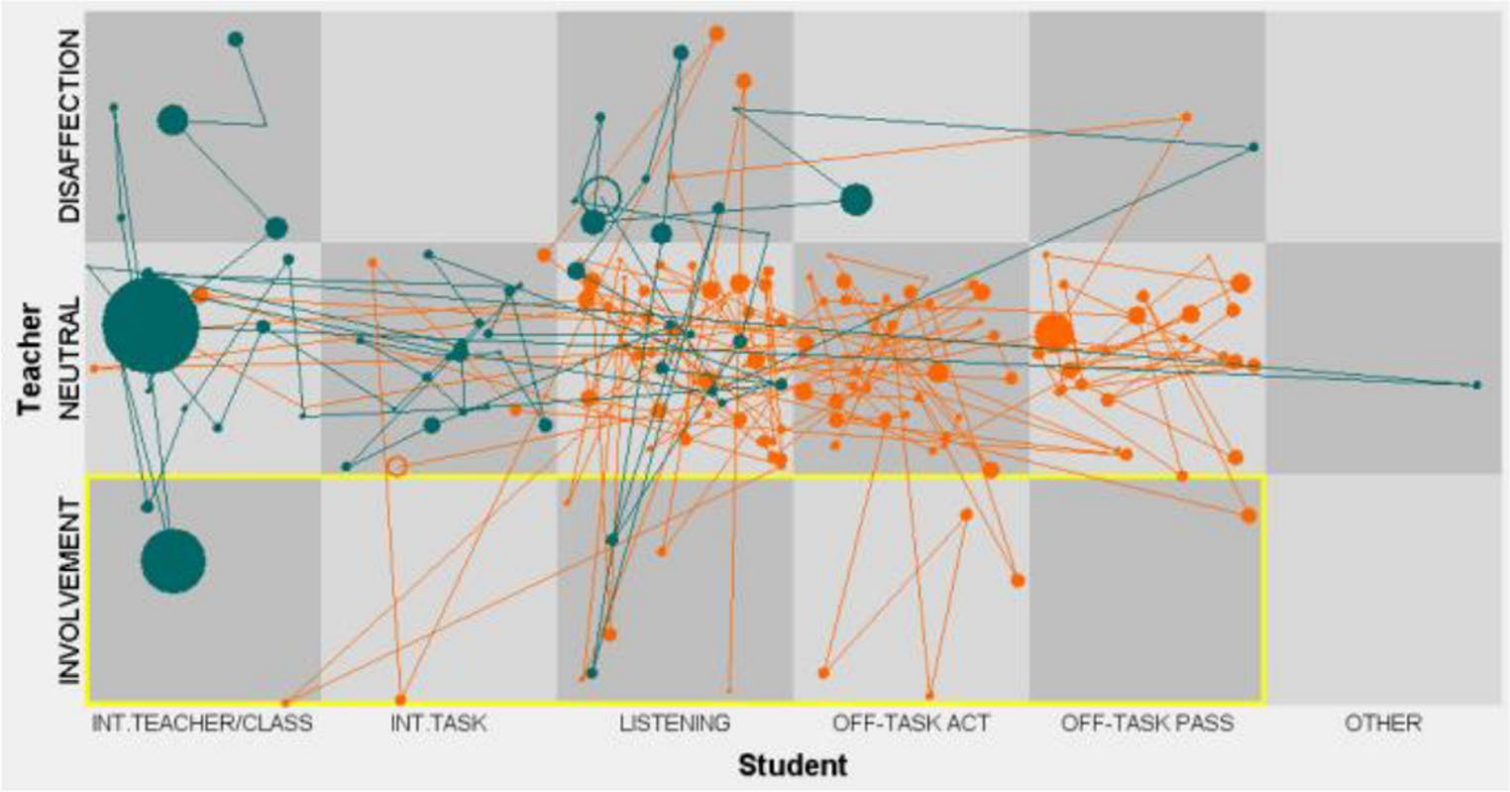
State Space Grids of lessons from Rafael and Cesar

‘Rafael’ provided a low proportion of involvement across his two Physics lessons in Mexico. Similar to the previous teacher-student pair (Anne and Alex), the interaction of ‘Rafael’ and student ‘Cesar’ visited all ‘desirable’ and ‘undesirable’ teacher and student states, making the interaction very flexible. Notably, the attractor state – the most observed pattern – for this teacher-student pair was the teacher being ‘Neutral’ while the student was ‘Listening’ (See Fig. 7).

When comparing the two dyads with high and low proportions of teachers’ involvement, we can observe that the teacher-student interactions were rather similar as they both visited all ‘undesirable’ and ‘desirable’ teacher and student states. Thus, in both examples there is great flexibility in the lessons.

### Similarities and Differences in the Most Frequent Teacher- Student Interaction Patterns Between the Netherlands and Mexico

**Fig. 8:**
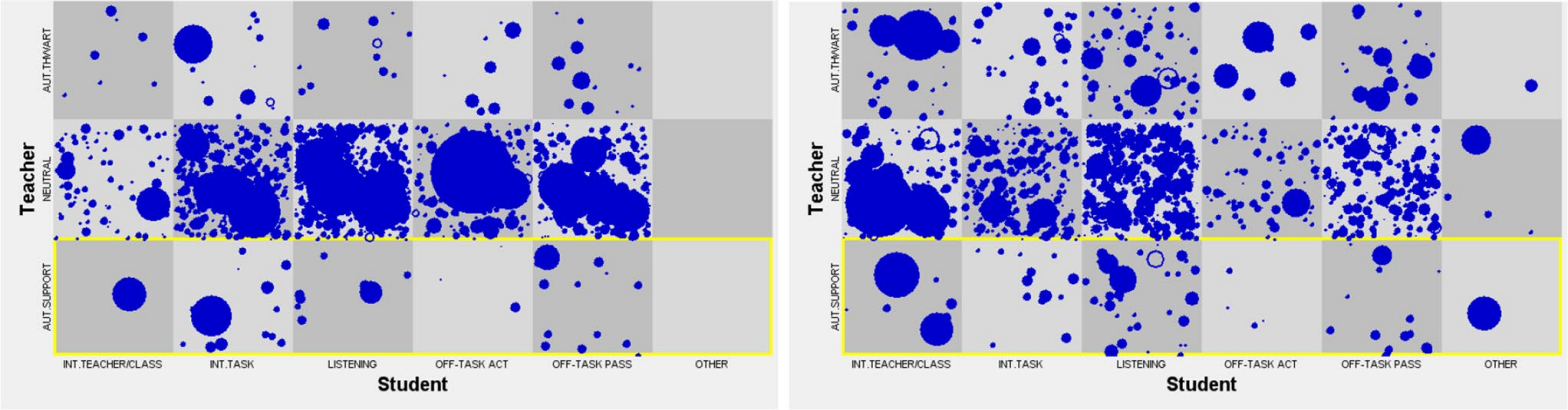
State Space Grids of autonomy-support across all lessons of all Dutch (left side) and Mexican (right side) participants. The vertical axis illustrates the teacher states: autonomy-support, neutral, and autonomy thwart. The horizontal axis shows all student states: ‘Engaged’ (interacting with teacher/class, interacting with a task, listening), ‘disengaged’ (off-task active, passive) and ‘other’. The region of interest (teachers provision of autonomy and the autistic students’ responses) is marked in yellow

When it comes to autonomy, overall, students were given few opportunities to make their own choices in the teacher-student interaction (see Fig. 8). This co-occurred with engaged and disengaged student states. Interestingly, teachers (and particularly the Dutch ones) stayed within the ‘Neutral’ state in the autonomy dimension. Yet, there was student variation, as students visited many states across the grid. Among Dutch and Mexican participants, the most frequently observed pattern was the teacher being ‘Neutral’ teacher while the student was ‘Listening’. Unfortunately, based on the number of visits, it was more common for teachers in both countries to thwart students’ autonomy than support it, with Mexican teachers being more pulled than the Dutch teachers to this state.

**Fig. 9:**
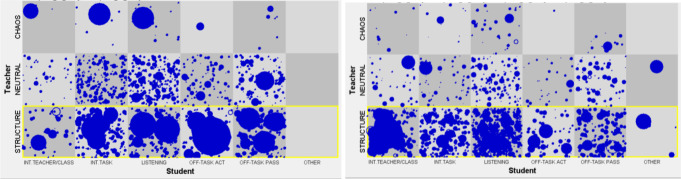
State Space Grids of structure across all lessons of all Dutch (left side) and Mexican (right side) participants. The vertical axis illustrates the teacher states: structure, neutral, and chaos. The horizontal axis shows all student states: ‘Engaged’ (interacting with teacher/class, interacting with a task, listening), ‘disengaged’ (Off-task active, passive) and ‘other’. The region of interest (teachers provision of structure and the autistic students’ responses) is marked in yellow

Autistic students regularly received step-by-step guidance during the teacher-student interaction. Notably, Dutch teachers were predominantly ‘Neutral’ while students were ‘Listening’, which is similar to the autonomy dimension. In contrast, the most frequent pattern observed in Mexican participants was the teacher giving ‘Structure’, which co-occurred with students ‘Listening’. Fortunately, teachers in both countries were more in a desirable state of providing ‘Structure’ rather than showing ‘Chaos’ (see Fig. 9).

**Fig. 10:**
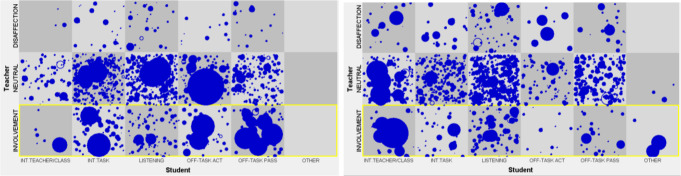
State Space Grids of involvement across all lessons of all Dutch (left side) and Mexican (right side) participants. The vertical axis illustrates the teacher states: involvement, neutral, and disaffection. The horizontal axis shows all student states: ‘Engaged’ (interacting with teacher/class, interacting with a task, listening), ‘disengaged’ (off-task active, passive) and ‘other’. The region of interest (teachers provision of involvement and the autistic students’ responses) is marked on yellow

Overall, teacher-student pairs moved across all states in the involvement dimension. Notably, Mexican teachers showed more disaffection than Dutch teachers. The predominant pattern in both countries was the teacher being ‘Neutral’ while the student was ‘Listening’ (see Fig. 10).

## Discussion

The present study used classroom observations and State Space Grids to analyze the interaction dynamics between teachers and autistic students in secondary education in the Netherlands and Mexico. Overall, these interactions were highly variable, as the dispersion values of most of the teacher-student interactions (across all dimensions) were between 0.6 and 0.9. This is in line with previous research indicating that the teacher-student interactions (of typically developing students) tend to move in-and-out from one state into another throughout a lesson (Smit et al., 2022).

In terms of autonomy support, we observed that students were provided with few opportunities to make choices. This is in line with findings from Heyworth et al. (2021) and Shea et al. (2013), who reported that the school environment does not often take into account the (autistic) students’ needs and interests. Previous studies with non-autistic participants have found autonomy-support to be a crucial factor influencing engagement (Snikkers-Mommer et al., 2024). Although teachers cannot be expected to be in an autonomy-supportive state all the time, our results seem to indicate that this would be a good addition to their repertoire of instructional strategies, as more desirable engagement states occurred in students when autonomy support was provided. However, teachers’ opportunities to provide autonomy support may be affected by specific barriers within different educational contexts, such as curriculum constraints (Emam & Farrell, 2009). Future studies should explore these contextual barriers in greater depth, to better understand the challenges teachers face and to develop targeted, practical recommendations that support the implementation of autonomy-supportive practices across diverse educational environments.

In terms of structure, many teachers in this study often provided step-by-step guidance to their students. We frequently observed that this co-occurred with students being engaged with a task or listening. In the teacher-student pair with a high proportion of structure, we also observed that students showed more engagement compared to the teacher-student pair with low levels of structure, in which the interactions oscillated between engaged and disengaged states. Interestingly, we noted that some dyads had a disengaged student state regardless of teachers’ frequent provision of structure. This counterintuitive finding may be explained by the fact that some teachers implement structure in a more controlling manner (Domen et al., 2020), or it may simply be due to the fact that autistic students have difficulty staying engaged for long periods of time (Zajic et al., 2020).

In terms of involvement, teachers displayed low levels of warmth and affection during their interactions. Regardless of whether students were provided with high or low proportions of involvement, the teacher-student interactions fluctuated between engaged and disengaged student states.

Even though our coding conventions were the same for all lessons, notably, the subject of a lesson (for example, Math, English, Sociology) can influence teacher-student interactions and the need-supportive strategies used in the moment (Maulana et al., 2012). For example, the highly structured Physics lesson from teacher ‘Rafael’ in the Mexican context—focused on problem-solving—offered frequent step-by-step guidance, resulting in greater student engagement. In contrast, the Sociology lesson from teacher ‘Cindy’ discussing societal issues, which did not have a hands-on task, involved less structure and corresponded with higher levels of student disengagement. A lesson that relies solely on listening and paying attention, without hands-on tasks may be problematic, as some autistic learners struggle to process large amounts of oral information (Carrington et al., 2003). Similarly, certain teaching activities may limit the extent of need-support teachers can provide to students. To prevent disengagement in (inherently) less-structured lessons and activities, it is highly recommended to provide visual support, such as video explanations, images, and pictograms. These supports can help autistic students better understand and identify key information about a topic (Esqueda Villegas et al., submitted).

Last, although the educational systems of the Netherlands and Mexico seemed rather different, we found many similarities in the interactional patterns. First, there was a tendency for teachers (in both countries) to be more controlling than autonomy supportive. This is worrisome given that autonomy-thwart can cause students’ disengagement (Cents-Boonstra et al., 2021; Ryan & Deci, 2018). Second, for many teacher-student pairs the most common interaction pattern was teacher providing ‘Structure’ and an engaged student state, such as ‘Listening’. Indeed, this may be beneficial to many autistic students who have strong preference for predictable and structured environments (Saggers et al., 2011). A third dominant pattern across the Dutch and Mexican teachers was to display few levels of involvement and remain more ‘Neutral’ during the teacher-student interaction. Similar to the autonomy dimension, although it is not expected for teachers to show involvement during the whole lesson, interactions that contain relatively high levels of involvement have more engaged student states. Therefore, these findings give an indication on how to improve students’ engagement and well-being.

### Strengths and Limitations

Although our study included only six teacher-student pairs in the Netherlands and seven in Mexico, it provides a valuable step towards understanding complex interaction processes in the classroom - especially given that much of the existing literature has relied on teacher or self-report measures to study the interactions of autistic students. Due to the in-depth nature of the interaction analysis offered by the state space grid technique, researchers have pointed out that studies using this method are generally considered reliable when conducted with smaller samples (e.g., Menninga et al., 2022). That being said, we did not consider some more distal factors that may influence the teacher-student interaction, such as years of teaching experience (Caplan et al., 2016; Feldman et al., 2019), teaching subject (Maulana et al., 2012) or when the data collection took place, such as the beginning or end of the school year (Smit et al., 2022). Moreover, we analyzed teacher-student behaviors that co-occurred. Therefore, these State Space Grids only considered immediate actions and not interaction patterns that are more complex, such as delayed responses or longer sequences of actions. Along the same line, the students’ behaviors during independent work may not be (fully) reflected in the State Space Grids. Lastly, although our coding scheme was based on previous research and demonstrated sufficient interrater reliability, it still presupposes an interpretation of teacher and student behavior. Notably, behaviors can be interpreted in different ways. For example, while we considered daydreaming to be “off-task passive behavior” in our study, other studies suggest that daydreaming can potentially be very useful for students’ comprehension (Soemer et al., 2019). Thus, future studies could provide an even more thorough analysis of individual student behavior, including students’ preferred ‘engaged’ behaviors during instruction.

## Conclusion

This is one of the first studies using real-time classroom observations and State Space Grids to analyze the teacher-(autistic) student interaction in secondary education. These types of studies are scarce (Esqueda Villegas et al., 2024), but provide many clues to the current and in-the-moment learning experiences of autistic students. We therefore encourage researchers in other countries to move beyond self-reports and questionnaires when studying teacher- (autistic) student interactions and instead incorporate classroom-based observational methods.

Our results show that there is great variation in the interaction between teachers and autistic students in secondary schools. However, Dutch and Mexican teachers seem to miss opportunities to provide explicit autonomy support; this contextual factor is minimally present in teacher-student interactions. This is consistent with previous research suggesting that the amount of autonomy support provided by teachers is not at a desirable level (Snikkers-Mommer et al., 2024). It was more common, however, for teachers in both countries, to provide their students with structure, which co-occurred (in most cases) with more engagement of autistic students. Yet, it should be noted that this desirable interaction pattern was not observed in *all* teacher-student pairs. Perhaps for these students, structure seems implemented in a more controlling form (Domen et al., 2020), or may simply not match their learning preferences. Since interaction patterns tend to stabilize across the school year, particular attention needs to be paid to disengaged student states that may be harder to break.

## Electronic supplementary material

Below is the link to the electronic supplementary material.

Supplementary Material 1

Supplementary Material 2
