# Supplementary material for: Teacher-Student Interactions of Autistic Adolescents: Relationships between Teacher Autonomy Support, Structure, Involvement and Student Engagement
Source: J Autism Dev Disord. 2025 Feb 14;56(7):2606–24. doi: 10.1007/s10803-025-06723-0 (PMC13346278; doi:10.1007/s10803-025-06723-0)
Supplement: Supplementary file 1 — Supplementary Material 1 [file 10803_2025_6723_MOESM1_ESM.docx]

Coding protocol

**The interaction between teachers and students with Autism Spectrum Disorder in mainstream secondary schools**

For this research, you will code the behavior/expressions of a teacher and a student with an autism spectrum disorder (ASD). Although we will “zoom in” on the interaction between the teacher and the autistic student, the teacher is likely to address the *whole class* the majority of time of the lesson and therefore, we will code this and not only one-to-one interaction (which might be scarce).

Each lesson is coded in six rounds. Round 1 is coding ***all*** teacher’s utterances during the lesson. This section focuses exclusively on what the teacher is saying to the whole class, a group of students to which the student with ASD belongs, or only at the student with ASD. That is to say, you will not focus on the verbal or non-verbal behavior of the student with ASD yet. The teacher’s utterances coded during round 1 will be used in rounds 2 to 5. Round 2 looks specifically at the degree of **autonomy-support/autonomy thwart** that the teacher provides during the lesson. Round 3 involves **structure/chaos** in the teacher’s behaviors. Round 4 relates to **involvement/disaffection** provided by the teacher. In round 5, the **non-verbal communication** of the lesson will be coded. Lastly, in round 6, the **behaviors/expressions of the student** with ASD are coded.

**Note on Mediacoder**

Mediacoder can be accessed using the following address: <https://mediacoder.gmw.rug.nl/>. There is a ‘Help’ option available for specific questions. Mediacoder works best with videos in the WebM format. Therefore, make sure you choose a WebM video when you click on “Open video file”.

After uploading a video, you are going to define your codeset. Then you start coding. Don’t forget to regularly save the file (“save mediacoder file”), to make sure you won’t lose any work!

**Round 1: Teacher’s utterances**

**Teacher codes**

**Teacher’s utterances.**

You code round 1 in the *Mediacoder* program. <https://mediacoder.gmw.rug.nl/>

In round 1 you code ***all*** verbal utterances of the teacher. These can be aimed at the entire class, the autistic student, or at a group of students to which the student with ASD belongs. Non-verbal utterances and background noises are not coded.

Decision rules

*Rule 1:* You code the teacher’s utterances during the whole lesson. As soon as a new utterance starts, drop the corresponding code “1”. When the utterance ends, put down the stop code “0”. There may be some time between the stop code “0” and a new code “1” for a new utterance, because, for example, the classroom is noisy or a student is talking. Make sure that you code the utterances and stop codes on time. If you are too late to realize an utterance is over (or too late to realize a new utterance has started), go back with the rewind button to a few seconds before.

*Rule 2:* You recognize the end of an utterance (and the start of a new one) after a brief pause in the teacher’s talk. To give a rough indication, an utterance after a 2-second pause marks a new utterance. To give an example:

**1:** OK everyone, I’d like to get started here, please take your…

**0:** 2 second pause, (because the teacher is briefly distracted by something)

**1:** Please take your book and open on page 9.

**0:** Pauses for a few seconds and waits for everyone to open the book

**1:** This week we start with a new chapter, focusing on algebra. You may recognize some of this from last year, but we haven’t covered this in much detail, which is what we’re going to do in the next few lessons. We start this lesson by a quick explanation about paragraph 1 and 2, then you’ll work independently for some time, and if we have some time toward the end, we make a quick start with paragraph 3.

**0:** Takes a breath, turns his back to the class and starts up digital blackboard

**1:** Ok, write along with me….

**0:** Pauses (writing on schoolboard)

As you can see, this means that some utterances are rather brief, while some others (the 3^rd^ utterance in this example) are a little longer.

*Rule 3:* You will code all teacher utterances when he or she addresses the whole class, for example during instruction time. During instruction time, you will also code an utterance if, for example, the teacher briefly says something to another student while he/she is explaining an assignment to the class.

However, during *independent work* (when the teacher walks around to answer questions), you do **not** code an utterance if the teacher is only targeting another student individually or a group of students to which the student with ASD does not belong. Tip: If you are not sure whether the student with ASD belongs to the group the teacher is talking to, it often helps to look further into the recording to see if this is the case.

**Codes to use for this round:**

**1 Teacher’s utterances**

**0 stop code**

- **Save the file as: Codingautism_round1_schoolpseudonym_teacherpseudonym_studentpseudonym_Lesson-number_name-of-coder**

Examples:

CodingAutism_round1_NorthernSecondarySchool_Peter_Maria_Lesson-1_Anne

CodingAutism_round1_NorthernSecondarySchool_Peter_Maria_Lesson-2_Anne

Note: Please follow this naming exactly, so that we always know which codes of which film it concerns.

- **Export file to Excel**

**Round 2: Autonomy support/autonomy thwart**

You code round 2 by adding codes A, B or C to the teacher’s utterances you coded during round 1.

*In this round you classify* ***all*** *the teacher’s utterances (coded in round 1) into three main categories; autonomy-support, autonomy thwart and autonomy support/autonomy thwart not visible or present.*

- **Open the file: Codingautism_round1_schoolpseudonym_teacherpseudonym_studentpseudonym_Lesson-number_name-of-coder**

**Codes to use for this round and examples**

| **A** | **Autonomy-support** |
| --- | --- |
| **B** | **Autonomy-thwart** |
| **C** | **Autonomy support/autonomy thwart not present or visible** |

**(A) Autonomy support**

Key elements: providing choice, fostering relevance, and taking the student’s perspective.

We code **A** when:

| The teacher allows the student to make decisions, such as where to sit or who to work with during group activities during the classroom | *“You can choose which paragraph you want to work on first”*  *“You can choose your own collaboration partner for this”*  *“Feel free to use your notes when working on this”*  *“You can use your calculator or do the calculations mentally”* |
| --- | --- |
| The teacher takes the student’s perspective | *“I see why you would do it like this”* |
| The teacher takes into consideration the ideas and suggestions from the student | *“Ok, so you prefer to have a short break in between, that’s fine with me”* |
| The teacher lets the student(s) know the importance of the tasks they will perform | *“You guys will be voting soon, next election. So, this issue of activism and review are very important”*  *“This will be on the exam, so we have to practice this today”* |

**(B) Autonomy thwart**

Key elements: control, forcing activities, and not taking the student’s perspective.

We code **B** when:

| The teacher uses controlling language | *“If you would quickly grab a seat. We don’t have a lot of time”*  *“Sit down now!”*  *“Start working now”* |
| --- | --- |
| The teacher sets strict rules or the pace in which the students have to work | *“I expect you to finish until the third paragraph” “Okay, if you wrap it up, please…three…two…one…”* |
| The teacher does not explain why the task is important or relevant | *“We have to do this today, period”* |
| Forces the student to do the activity | *“No, we won’t work on our personal project today, we will focus on chapter 4”* |

**(C) Autonomy support/autonomy thwart not present or visible**

There may be many utterances that are *neutral* and do not fall under the category autonomy support (A) or autonomy thwart (B). You will code these as autonomy support/autonomy thwart not present or visible (C). For example, if the teacher’s utterance is related to communicating clear and detailed instructions, this should be coded as *structure* (D) in round 3. Therefore, not all utterances are expected to “fit” the codes A and B from round 2.

Note: it is possible that an utterance falls into two categories from different rounds of coding. For example, the utterance *“we are going to vote. You have to vote for one. So, not voting is not a choice. You can’t vote twice”* can be coded in this round as autonomy thwart (B) because the student is not allowed to choose more than one option and has to vote. Yet, the same utterance is communicating clearly what the student has to do (select only one option). Therefore, the utterance also falls under the category of “structure” in round 3. This is the main reason why you will work with ***all*** teacher’s utterances (coded in round 1) in rounds 2, 3 and 4. In this round, however, you only focus on which utterances fit the descriptions of codes A to C.

- **Save the file as: Codingautism_round2_schoolpseudonym_teacherpseudonym_studentpseudonym_Lesson-number_name-of-coder**

Examples:

Codingautism_round2_NorthernSecondarySchool_Peter_Maria_Lesson-1_Anne

Codingautism_round2_NorthernSecondarySchool_Peter_Maria_Lesson-2_Anne

Note: Please follow this naming exactly, so that we always know which codes of which film it concerns.

**Round 3: Structure and chaos**

You code round 3 by adding codes D, E or F to the teacher’s utterances you coded during round 1.

In this round, you classify **all** the teacher’s utterances into three main categories; structure, chaos, and structure/chaos not visible or present.

- **Open the file: Codingautism_round1_schoolpseudonym_teacherpseudonym_studentpseudonym_Lesson-number_name-of-coder**

**Codes to use for this round and examples**

| **D** | **Structure** |
| --- | --- |
| **E** | **Chaos** |
| **F** | **Structure/chaos not present or visible** |

**(D) Structure**

Key elements: Clarity, guidance (scaffolding), positive feedback about the task, or informational feedback when something is incorrect. Note: Informative feedback informs learners *why* their responses are correct or incorrect, providing with task-related information.

We code **D** when:

| The teacher is explicit with regard to what the students have to learn, what they have to do and how | *“We’re going to start with Question 1 on page 6”*  *“We have 30 minutes for this part”*  *“Today we are going to learn how to…”* |
| --- | --- |
| The teacher provides step by step directions on what the student has to do during an on-going activity | *“Remember, always start by writing the brackets in the right places”*  *“Now, let’s take a look into exercise 2 and what we have to do to answer questions 3 to 5”*  *“The first thing we need to write on a sentence is…and then…”* |
| The teacher gives verbal information to the students about rules, agreements or what kind of behavior s/he expects from them | *“Please be silent while I explain what you have to do on exercise 3”*  *“Now, go and sit next to your partner”*  *“You can start working on your own and in silence”* |
| The teacher provides verbal information about different parts of the lesson or discusses previous lessons, topics or assignments | *“Okay. I am going to start now. Today we are going to talk about…”*  *“Last week I explained to you that…”*  *“We have already learned, a couple of lessons ago, that…”* |
| The teacher discusses what they learned or did during the lesson and provides instructions for what follows next, such as homework or the topics for the next lesson | *“For next class, I’d like you to bring two examples of…”*  *“Today we discussed different ways in which we can solve an equation. For next class, I would like for you to…”*  *“So, your homework will be answering questions 3 and 4 of page 37 from your exercise book”* |
| When the student is doing a good job, or has a good attitude towards work, the teacher notices it an lets him/her know | *“You were all very focused, good!”*  *“A lot of good questions were asked”* |
| The teacher gives information about the time the students have for an activity | *“You have three more minutes”*  *“I will give you five minutes to work on exercises 4 and 5”* |
| When the student is incorrect, the teacher helps by giving **informative** feedback | *“No, wait a minute, did you look at the brackets”*  *“Yes, but remember when there is a minus sign without a number, we should read it as minus 1”* |

Note: If the teacher’s utterance addresses a concept, theory or provides an example without explicitly connecting the new knowledge to a previous/future lesson, we ***do not*** code this as structure.

**(E) Chaos**

Key elements: no clarity, no guidance, negative feedback about the task, or evaluative feedback. Note: Evaluative feedback provides whether the student did something correct or not, but does not convey the information and guidance that students can use to improve.

We code **E** when:

| The instructions provided by the teacher are confusing and unclear | *“You can do part A or part B, maybe we get to the explanation of part C, but we have to see, maybe not”* |
| --- | --- |
| The teacher provides negative or purely evaluative feedback when the student does not have the right answer | *“No. That is not the answer I was looking for”*  *“No. It is wrong. That shouldn’t be the result”*  *“Do it again. Your answer is wrong”* |
| The teacher uses verbal behavior to punish the student | *“You were not paying attention, weren’t you?”*  *“You have the instructions in your book, look into that”* |

**(F) Structure/chaos not present or visible**

If a teacher’s utterance does not fall under the category structure (D) or chaos (E), you will code it as structure/chaos not present or visible (F). It might be that this *neutral* utterance falls under another category from a different round of coding. Therefore, not all utterances are expected to “fit” the codes D and E from round 3.

Reminder: it is possible that an utterance falls into two categories from different rounds of coding. For instance, the utterance *“you have the instructions in your book, look into that [with a stern tone]”* in this round is coded as chaos (E). Yet, this utterance also displays disaffection towards the student because the teacher is addressing him/her with an unfriendly tone. Therefore, this should be coded as disaffection (H) in round 4. This is the main reason why you will work with ***all*** teacher’s utterances (coded in round 1) in rounds 2, 3 and 4. In this round, however, you only focus on which utterances fit the descriptions of codes D to F.

- **Save the file as: Codingautism_round3_schoolpseudonym_teacherpseudonym_studentpseudonym_Lesson-number_name-of-coder**

*Examples:*

Codingautism_round3_NorthernMiddleSchool_Peter_Maria_Lesson-1_Anne

Codingautism_round3_NorthernMiddleSchool_Peter_Maria_Lesson-2_Anne

Note: Please follow this naming exactly, so that we always know which codes of which film it concerns.

**Round 4: Involvement/disaffection**

You code round 4 by adding codes G, H or I to the teacher’s utterances you coded during round 1.

*In this round you classify* ***all*** *the teacher’s utterances (coded in round 1) into three main categories; involvement, disaffection, or involvement/disaffection not visible or present.*

*Decision rules*

If an utterance contains elements of both involvement and disaffection, and you are unsure which code to assign it, take a closer look into the whole utterance and especially at the ending of it. Examples:

*“Well, you are making a lot of progress today!* ***Quite unusual from you!”***

*“You really put effort into your homework.* ***Didn’t you?”***

Although the first parts of these examples could be considered involvement (since the teacher seems to be praising the student) the ending of the utterances show the contrary: the teacher is mostly being sarcastic and displaying disaffection towards the student. Therefore, it is essential to “look further” into the context of the utterance and/or the tone of it, if it seems to be a mixture of both involvement (G) and disaffection (H).

- **Open the file: Codingautism_round1_schoolpseudonym_teacherpseudonym_studentpseudonym_Lesson-number_name-of-coder**

**Codes to use for this round and examples**

| **G** | **Involvement** |
| --- | --- |
| **H** | **Disaffection** |
| **I** | **Involvement/disaffection not present or visible** |

**(G) Involvement**

Key elements: affection, attunement, dedication of resources and time, dependability.

We code **G** when:

| The teacher addresses students with a friendly tone, shows concern | *“I hope you feel better again today”*  *“Do not be worried about the exam. It will cover topics that we’ve already looked into”* |
| --- | --- |
| The teacher is approachable and available for the students | *“Anybody else has a question about this?”*  *“Are there any questions about the topic we saw las class?”* |
| The teacher shows understanding of what the student finds important for him/her | *“I will grade your tests as soon as possible”*  *“I understand that you want to have a break so let’s finish checking this together”* |
| The teacher is caring and supportive towards the student(s) | *“Are you okay?”*  *“Do you need any help?*  *“Is the topic clear or would you like us to revise it together?”* |

**(H) Disaffection**

Key elements: rejection, no attunement, no dedication of resources, no dependability.

We code **H** when:

| The teacher talks to the student in an unfriendly tone and treats him/her unfriendly | *“You have the instructions in your book, look into that [with a stern tone]”*  *“Pay attention. You have the answer in your book”* |
| --- | --- |
| The teacher is not available when the student looks up for him/her | *“No [student’s name], we do not have time for questions”*  *“Go sit down. Don’t keep asking me”; “Do not ask me about that topic”* |
| The teacher is not understanding of the student’s needs | *“I do not want to hear any complaints, just start doing the activity”* |

**(I) Involvement/disaffection not present or visible**

If a teacher’s utterance is *neutral* and does not fall under the category involvement (G) or disaffection (H), you will code it as involvement/disaffection not present or visible (I). It might be that the utterance falls under another category from a different round of coding. Therefore, not all utterances are expected to “fit” the codes G and H from round 4.

Reminder: it is possible that an utterance falls into two categories. This is the main reason why you will work with ***all*** teacher’s utterances (coded in round 1) in rounds 2, 3 and 4.

- **Save the file as: Codingautism_round4_schoolpseudonym_teacherpseudonym_studentpseudonym_Lesson-number_name-of-coder**

Examples:

Codingautism_round4_NorthernSecondarySchool_Peter_Maria_Lesson-1_Anne

Codingautism_round4_NorthernSecondarySchool_Peter_Maria_Lesson-2_Anne

Note: Please follow this naming exactly, so that we always know which codes of which film it concerns.

**Round 5: Non-verbal aspects of the lesson**

In this round of coding, we look into the non-verbal behaviors of the lesson, such as facial expressions (J), gestures (K), and intonations (L).

Note: We will add these codes to the teachers’ utterances you coded in round 1. This means that we will lose some of the nonverbal gestures, but most of the time a gesture will be made with a verbal utterance present, and starting from the codes in round 1 is simpler and quicker.

**Codes to use for this round and examples**

| **J** | **Facial expression** |
| --- | --- |
| **K** | **Gesture** |
| **L** | **Intonation** |

**“J” Facial expressions**

Key elements: The teacher uses facial expressions that show emotions such as joy, anger, fear, disgust, sadness, or surprise.

Examples of facial expressions: The teacher smiles when the student gives the correct answer. The teacher raises/lowers his/her eyebrows to show surprise that a student knew the answer to the question. The teacher says to a student ‘stop talking’ while frowning his eyebrows.

Note: you may not always see the expression. We only code expressions that are very clear to us. It is inevitable that we would then miss some of the more subtle facial expressions, but we have to make sure we do this in a reliable way.

**“K” Gesture**

You will code K when the teacher uses a gesture *with a clear purpose.* The gesture, for instance, can intend to let the student know he is doing well, make something clearer or to emphasize a point to the class. *You do not use code “K” when the gesture has no specific purpose/intention. For example, if the teacher raises his arms to stretch, or to lean against the wall, or uses his hands while he/she is talking without conveying meaning with these movements.*

Examples of gestures:

- The teacher says, “Look at the board” and points at it with his finger.
- The teacher gives thumbs up to the student because s/he is doing a good job.
- The teacher has his arms crossed as he waits for the class to be silent.
- The teacher gives a 'wink' to the class when they give the correct answer.
- The teacher is nodding to let the student know he is listening or to indicate that an answer is correct.
- The teacher pats the back of the student to let him/her know s/he did a good job.
- The teacher raises one finger to indicate this is exercise 1; this is followed by a second finger, which indicates this is now exercise two.

**“L” Intonation**

You will code L if the teacher (during the utterance) increases or decreases his/her tone of voice and/or starts speaking faster/slower.

Examples of intonation:

- The teacher raises his/her voice to make it clear that an answer is correct.
- The teacher speeds up his explanation because he gets frustrated or s/he is running out of time.
- The teacher slows down to make something clear or emphasize a point to the class.
- The teacher starts to speak slowly so the student(s) can understand what they have to do during a task.

Note: you may not always notice changes in intonation. We only code intonation changes that are very clear to us. It is inevitable that we would then miss some of the more subtle uses of intonation, but we have to make sure we do this in a reliable way.

- **Save the file as: Codingautism_round5_schoolpseudonym_teacherpseudonym_studentpseudonym_Lesson-number_name-of-coder**

Examples:

Codingautism_round5_WestviewMiddleSchool_Peter_Maria_Lesson-1_Fernanda

Codingautism_round5_WestviewMiddleSchool_Peter_Maria_Lesson-2_Fernanda

Note: Please follow this naming exactly, so that we always know which codes of which film it concerns.

**Round 6: Student with ASD-related codes**

**Determining the behaviors of the autistic student**

In this round of coding, you will look at the behavior of the autistic student by focusing at the student's engagement in the lesson. For this purpose, we use the recordings that are aimed at the whole class (students’ camera).

*Decision rules*

All verbal utterances by the student are coded. Due to their social and communicational impairments, verbal utterances might be scarce. Therefore, we will also take into account non-verbal behavior: the student's intonation, facial expression, physical activity, viewing direction and verbal expression.

**Codes to use for this round and examples**

| **M** | **Actively interacting with the teacher and/or the class** |
| --- | --- |
| **N** | **Actively interacting with a task** |
| **O** | **On-task listening and paying attention** |
| **P** | **Off-task active** |
| **Q** | **Off-task passive** |
| **R** | **Other** |

**“M” ACTIVELY INTERACTING WITH THE TEACHER AND/OR THE CLASS**

The student is actively involved and interacting with the teacher. The student with ASD contributes to the lesson on his/her own initiative. The student shares with the teacher and the class his/her ideas or findings.

Signs: *Raising hand, eye contact with the teacher, responsive to the teacher, little distraction,*

Examples: *The student raises his/her hand to answer the teacher’s question or volunteers to go to the whiteboard. The student asks questions to understand the task or to provide a more comprehensive explanation of what the teacher has said.*

**“N” ACTIVELY INTERACTING WITH A TASK**

The student is involved in a given task and seems to be concentrated and interested in it. This code is related to doing an activity in his/her notebook, working on a problem or reading. This behavior can occur, for instance, while the teacher is giving instructions or discussing the content of the lesson. The student does **not** verbalize anything here.

Signs: *Not easily distracted by others, eye gazed focused on the board or book, staring at the task materials so to not miss any details, taking notes.*

Examples: *The student is reading a text during independent work. The student is working on a problem and writes down notes.*

**“O” ON-TASK LISTENING AND PAYING ATTENTION**

While the teacher is explaining the lesson/activity, the student with ASD is listening and paying attention. If a classmate is making an on-task contribution, the student with ASD pays attention as well.

Signs: *Eye gazed focused on the teacher or the student who is speaking.*

Examples: *The student is looking at the teacher or whiteboard, who is now providing instruction on what they have to do. A classmate asks a question to the teacher; the student with ASD turns around to look at the student who is speaking and turns back to the teacher when he/she starts to clarify the instructions.*

**“P” OFF-TASK ACTIVE**

The student with ASD **initiates** the off-task behavior by disrupting a classmate (who can be either participating in the lesson or working independently) or by interrupting the teacher with a non-academic question or comment. The student can also be manipulating objects that are not needed for the task.

Signs: *Focused on off-task behaviors, joins off-topic conversations, interruptions, grabs materials that are not used during the activity.*

Examples: *The student initiates a conversation that is not related to academic-issues with another student: “Have you seen movie X?” “What did you have for dinner yesterday?”. The student interrupts the teacher with a comment that is not related to the task: “Teacher? Yesterday I went fishing”. The student starts playing with his/her calculator, although this material is not needed for the task. The student hears other students talk about a TV show; s/he joins the conversation and stops paying attention to the task. The student empties and cleans his/her pencil case.*

**“Q” OFF-TASK PASSIVE**

The student is not at all focused or barely focused on the given task. The student appears to be daydreaming or is listening to a classmate’s off-task contribution. There is a certain amount of disinterest.

Signs: *Daydreaming behavior, procrastination, sighing, easily distracted, gaze on other students that are not working on the task.*

Example: *The student reads part of the instruction but is easily distracted by what is happening around him. The student turns his/her face and proceeds to pay attention to what another classmate is saying about a videogame.*

Note: code “Q” may be hard to see. If you are in doubt between code “Q” or N/O, we give the student the benefit of the doubt and code N/O.

**“R” OTHER**

We select this code when the student’s behavior does not fall within any of the previous categories. For example: the student momentarily leaves the classroom to go to the toilet or because s/he completed the activity they were working on.

This code is also used when the student is standing in line for the teacher to check the given task (as long as the student is *NOT* interacting with another classmate or with the teacher while waiting for their turn).

- **Save the file as: Codingautism_round6_schoolpseudonym_teacherpseudonym_studentpseudonym_Lesson-number_name-of-coder**

Examples:

Codingautism_round6_NorthernSecondarySchool_Peter_Maria_Lesson-1_Anne

Codingautism_round6_NorthernSecondarySchool_Peter_Maria_Lesson-2_Anne

Note: Please follow this naming exactly, so that we always know which codes of which film it concerns.

**Literature used for the coding protocol**

Christenson, S. L., Reschly, A. L., & Wylie, C. (2012). *Handbook of Research on Student Engagement*. Springer New York. <https://doi.org/10.1007/978-1-4614-2018-7>

Jang, H., Reeve, J., & Deci, E. (2010). Engaging students in learning activities: it is not autonomy support or structure but autonomy support and structure. *Journal of Educational Psychology*, *102*, 588 - 600. <https://doi.org/s://doi.org/10.1037/a0019682>

Loopers, J. H. (2022). *Unravelling the dynamics of intrinsic motivation of students with and without special educational needs.* [Thesis fully internal (DIV), University of Groningen].

Reeve, J., & Jang, H. (2006). What teachers say and do to support students' autonomy during a learning activity. *Journal of Educational Psychology*, *98*, 209-218.

Ryan, R. M., & Deci, E. L. (2000). Self-determination theory and the facilitation of intrinsic motivation, social development, and well-being. *Am Psychol*, *55*(1), 68-78. <https://doi.org/10.1037//0003-066x.55.1.68>

Ryan, R. M., & Deci, E. L. (2018). *Self-Determination Theory: Basic Psychological Needs in Motivation, Development, and Wellness*. Guilford Publications. <https://books.google.de/books?id=th5rDwAAQBAJ>

Skinner, E. A., & Belmont, M. (1993). Motivation in the classroom: Reciprocal effects of teacher behavior and student engagement across the school year. *Journal of Educational Psychology*, *85*, 571-581.

Stroet, K. (2014). *Studying motivation in classrooms: effects of teaching practices on early adolescents' motivation.* [Thesis fully internal (DIV), University of Groningen].

Stroet, K., Opdenakker, M.-C., & Minnaert, A. (2013). Effects of need supportive teaching on early adolescents’ motivation and engagement: A review of the literature. *Educational Research Review*, *9*, 65-87. <https://doi.org/https://doi.org/10.1016/j.edurev.2012.11.003>
