# Supplementary material for: Teacher-Student Interactions of Autistic Adolescents: Relationships between Teacher Autonomy Support, Structure, Involvement and Student Engagement
Source: J Autism Dev Disord. 2025 Feb 14;56(7):2606–24. doi: 10.1007/s10803-025-06723-0 (PMC13346278; doi:10.1007/s10803-025-06723-0)

**Supplementary Materials – State Space Grids**

Contents

[State Space Grids for (teacher) Autonomy-support and student (dis)engagement 3](#_Toc167437016)

[Richard and David – Lessons 1 and 2 4](#_Toc167437017)

[Richard and Alan – Lessons 1 and 2 4](#_Toc167437018)

[Maria and Rachel – Lessons 1 and 2 5](#_Toc167437019)

[Anne and Alex – Lessons 1 and 2 5](#_Toc167437020)

[Jack and Sandra – Lessons 1 and 2 6](#_Toc167437021)

[Henry and Simon – Lessons 1 and 2 6](#_Toc167437022)

[Rafael and Sebastian – Lessons 1 and 2 7](#_Toc167437023)

[Rafael and Cesar – Lessons 1 and 2 7](#_Toc167437024)

[Erick and Adrian – Lessons 1 and 2 8](#_Toc167437025)

[Erick and Daniel – Lessons 1 and 2 8](#_Toc167437026)

[Cindy and Alberto – Lessons 1 and 2 9](#_Toc167437027)

[Cindy and Jesus – Lessons 1 and 2 9](#_Toc167437028)

[Sofia and Sara – Lessons 1 and 2 10](#_Toc167437029)

[State Space Grids for (teacher) Structure and student (dis)engagement 11](#_Toc167437030)

[Richard and David – Lessons 1 and 2 12](#_Toc167437031)

[Richard and Alan – Lessons 1 and 2 12](#_Toc167437032)

[Maria and Rachel – Lessons 1 and 2 13](#_Toc167437033)

[Anne and Alex – Lessons 1 and 2 13](#_Toc167437034)

[Jack and Sandra – Lessons 1 and 2 14](#_Toc167437035)

[Henry and Simon – Lessons 1 and 2 14](#_Toc167437036)

[Rafael and Sebastian – Lessons 1 and 2 15](#_Toc167437037)

[Rafael and Cesar – Lessons 1 and 2 15](#_Toc167437038)

[Erick and Adrian – Lessons 1 and 2 16](#_Toc167437039)

[Erick and Daniel – Lessons 1 and 2 16](#_Toc167437040)

[Cindy and Alberto – Lessons 1 and 2 17](#_Toc167437041)

[Cindy and Jesus – Lessons 1 and 2 17](#_Toc167437042)

[Sofia and Sara – Lessons 1 and 2 18](#_Toc167437043)

[State Space Grids for (teacher) involvement and student (dis)engagement 19](#_Toc167437044)

[Richard and David – Lessons 1 and 2 20](#_Toc167437045)

[Richard and Alan – Lessons 1 and 2 20](#_Toc167437046)

[Maria and Rachel – Lessons 1 and 2 21](#_Toc167437047)

[Anne and Alex – Lessons 1 and 2 21](#_Toc167437048)

[Jack and Sandra – Lessons 1 and 2 22](#_Toc167437049)

[Henry and Simon – Lessons 1 and 2 22](#_Toc167437050)

[Rafael and Sebastian – Lessons 1 and 2 23](#_Toc167437051)

[Rafael and Cesar – Lessons 1 and 2 23](#_Toc167437052)

[Erick and Adrian – Lessons 1 and 2 24](#_Toc167437053)

[Erick and Daniel – Lessons 1 and 2 24](#_Toc167437054)

[Cindy and Alberto – Lessons 1 and 2 25](#_Toc167437055)

[Cindy and Jesus – Lessons 1 and 2 25](#_Toc167437056)

[Sofia and Sara – Lessons 1 and 2 26](#_Toc167437057)

# State Space Grids for (teacher) Autonomy-support and student (dis)engagement

## Richard and David – Lessons 1 and 2


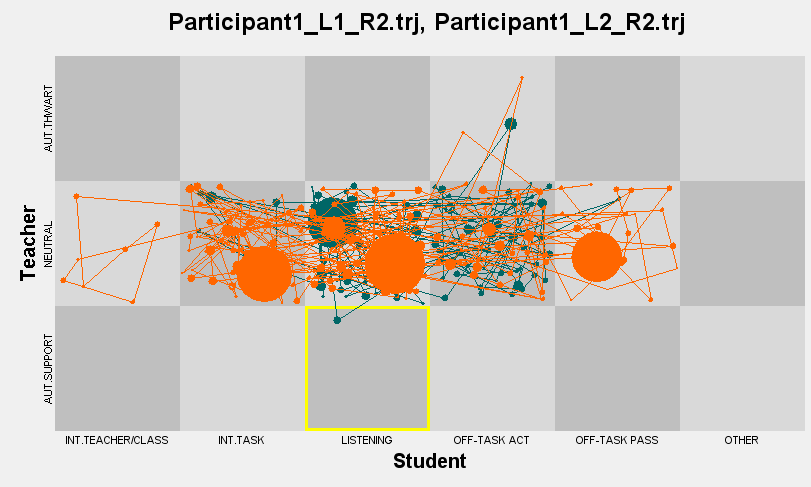


## Richard and Alan – Lessons 1 and 2


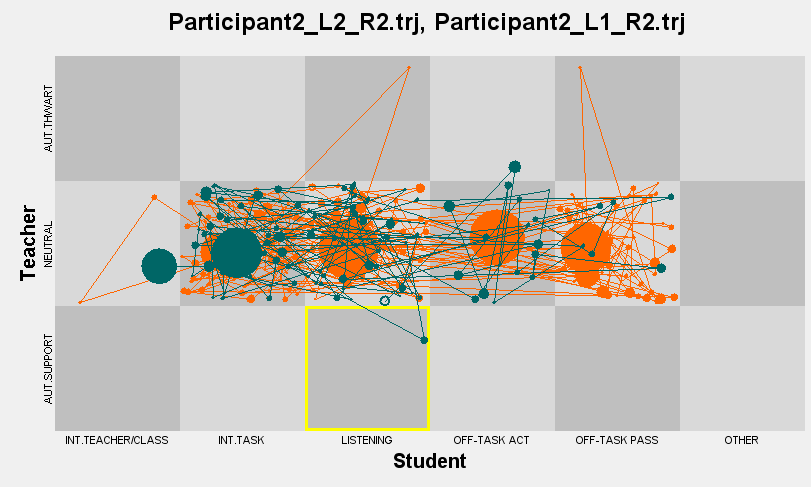


Note: The vertical axis illustrates the teacher states in the ‘autonomy’ dimension: autonomy-support, neutral and autonomy thwart. The horizontal axis shows all student states, which can be ‘engaged’ (interacting with teacher/class, interacting with a task, listening), ‘disengaged’ (active, passive) or ‘other’. The region of interest (teacher providing autonomy and the student’s co-occurring behavior) is marked in yellow. The blue color represents Lesson 1, whereas the orange color denotes Lesson 2.

## Maria and Rachel – Lessons 1 and 2


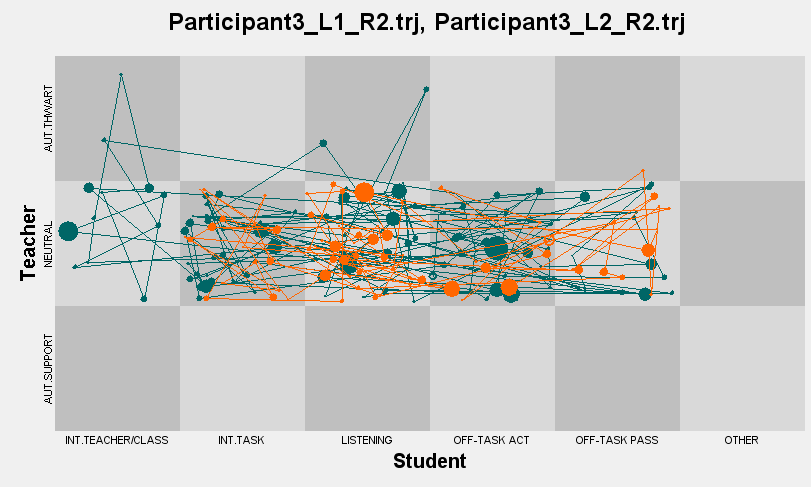


## Anne and Alex – Lessons 1 and 2


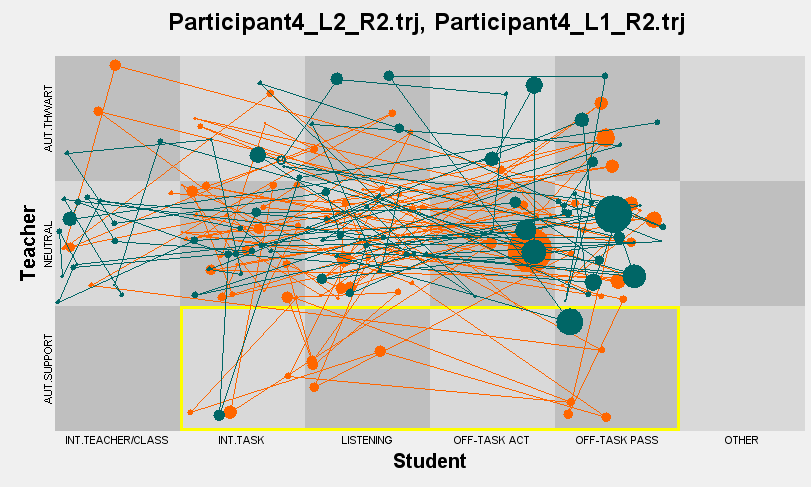


## Jack and Sandra – Lessons 1 and 2


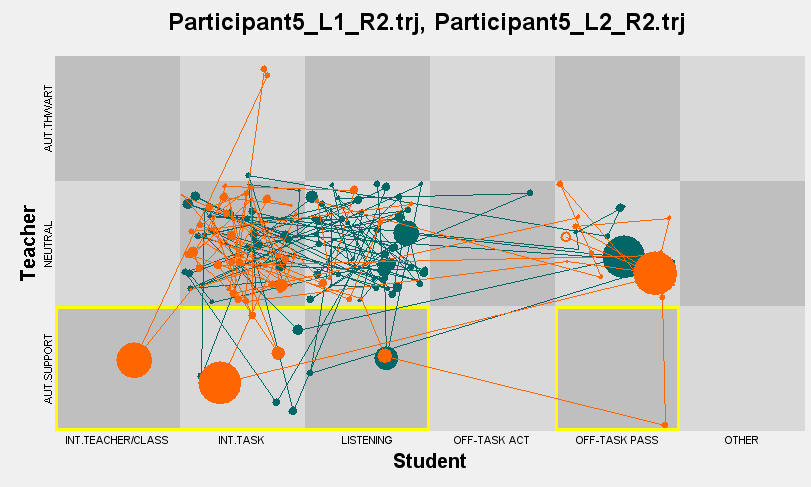


## Henry and Simon – Lessons 1 and 2


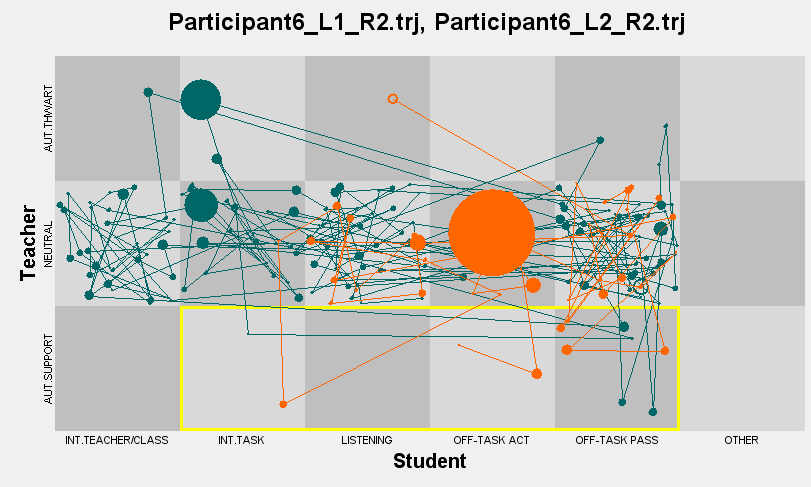


## Rafael and Sebastian – Lessons 1 and 2


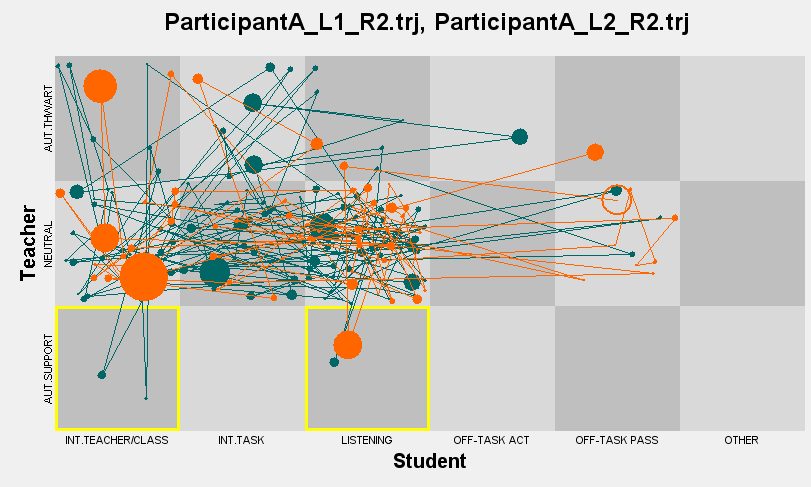


## Rafael and Cesar – Lessons 1 and 2


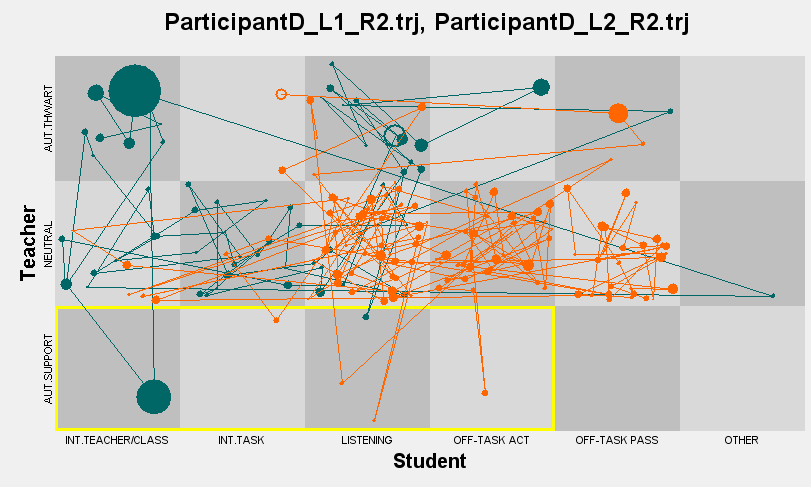


## Erick and Adrian – Lessons 1 and 2


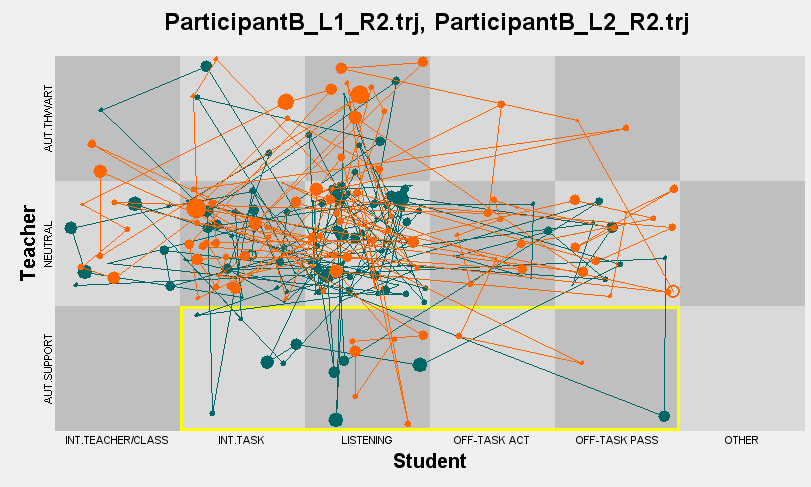


## Erick and Daniel – Lessons 1 and 2


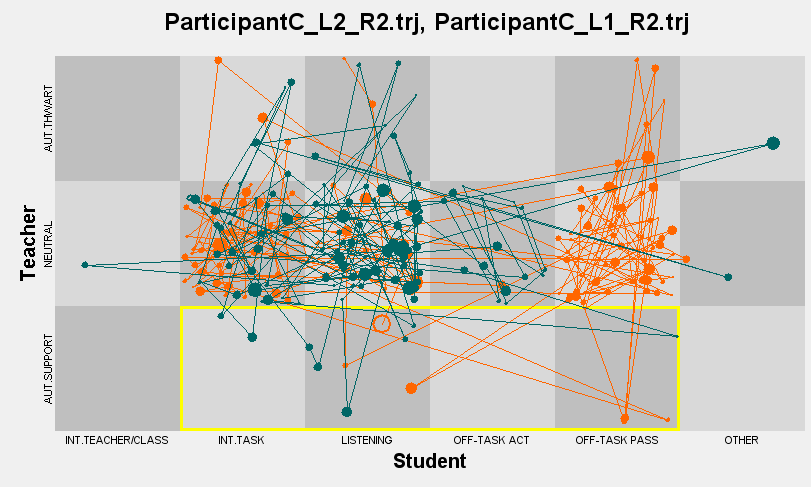


## Cindy and Alberto – Lessons 1 and 2


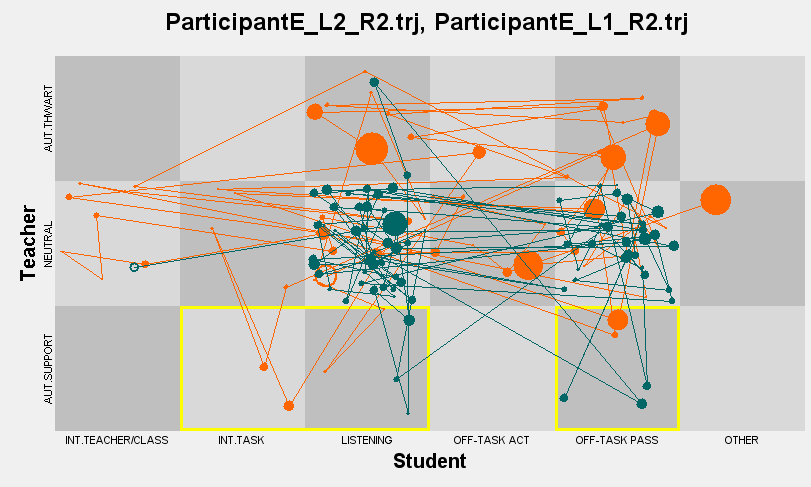


## Cindy and Jesus – Lessons 1 and 2


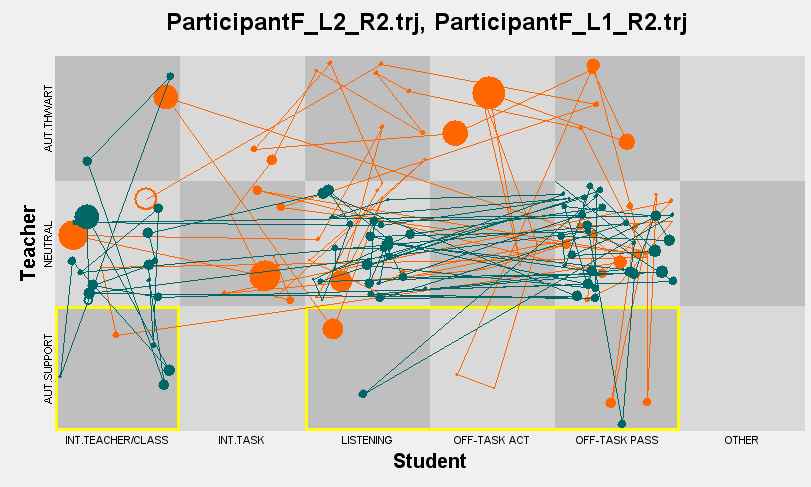


## Sofia and Sara – Lessons 1 and 2


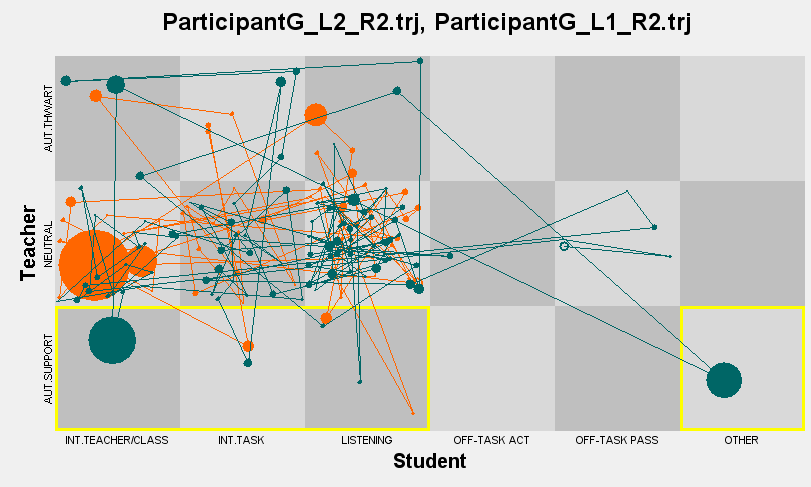


# State Space Grids for (teacher) Structure and student (dis)engagement

## Richard and David – Lessons 1 and 2


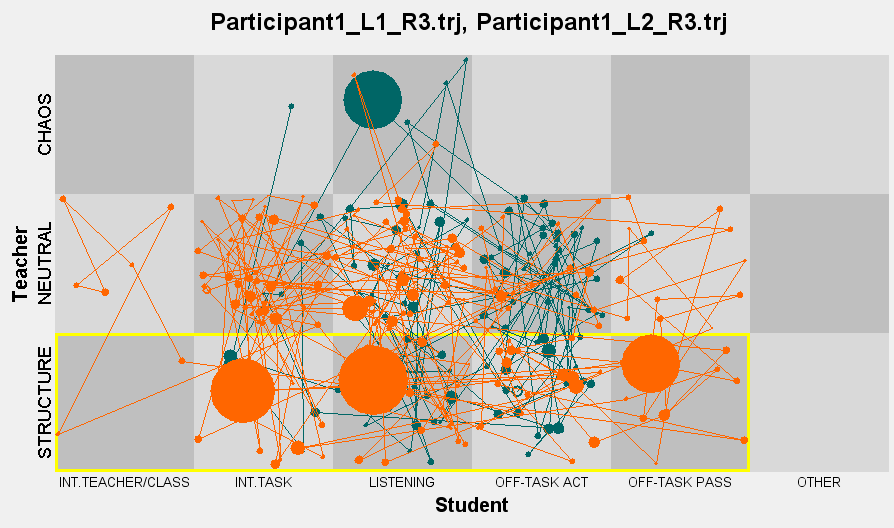


## Richard and Alan – Lessons 1 and 2


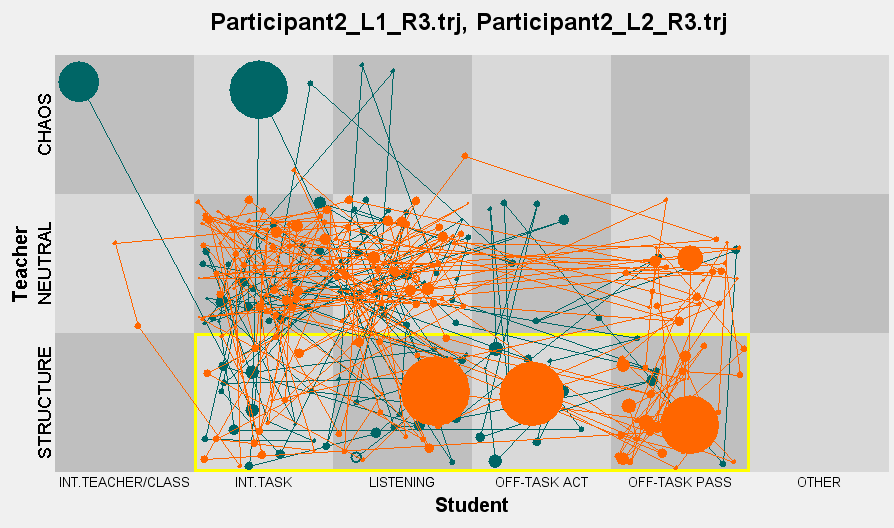


Note: The vertical axis illustrates the teacher states in the ‘structure’ dimension: structure, neutral and chaos. The horizontal axis shows all student states, which can be ‘engaged’ (interacting with teacher/class, interacting with a task, listening), ‘disengaged’ (active, passive) or ‘other’. The region of interest (teacher providing structure and the student’s co-occurring behavior) is marked in yellow. The blue color represents Lesson 1, whereas the orange color denotes Lesson 2.

## Maria and Rachel – Lessons 1 and 2


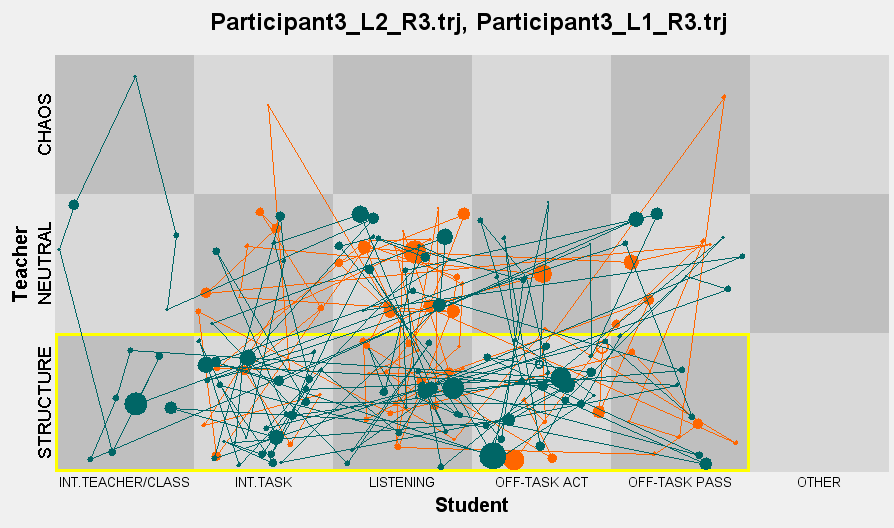


## Anne and Alex – Lessons 1 and 2


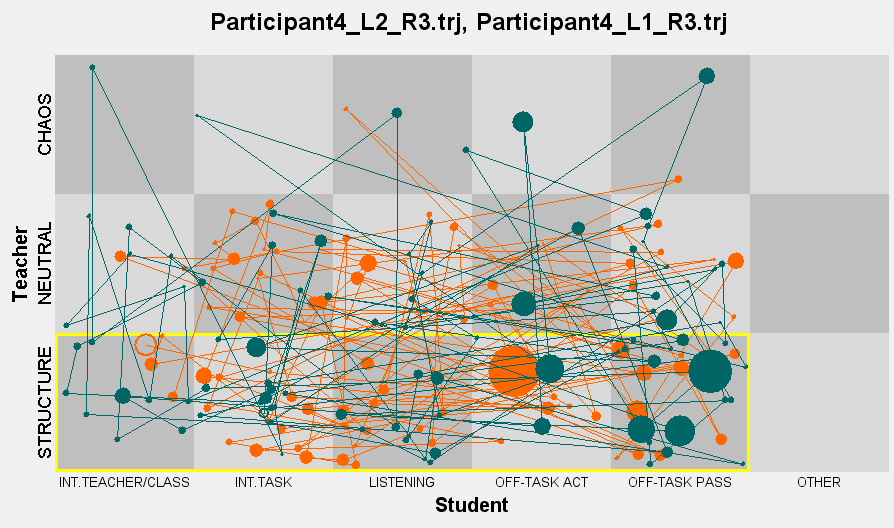


## Jack and Sandra – Lessons 1 and 2


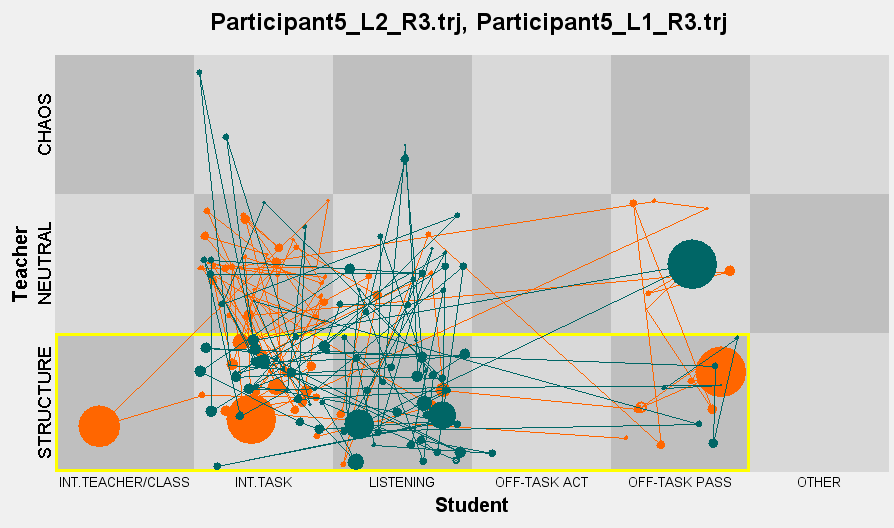


## Henry and Simon – Lessons 1 and 2


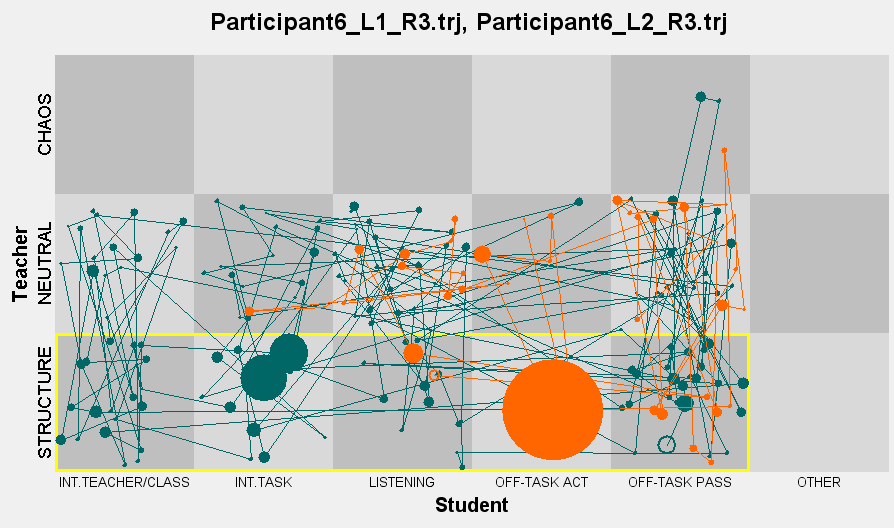


## Rafael and Sebastian – Lessons 1 and 2


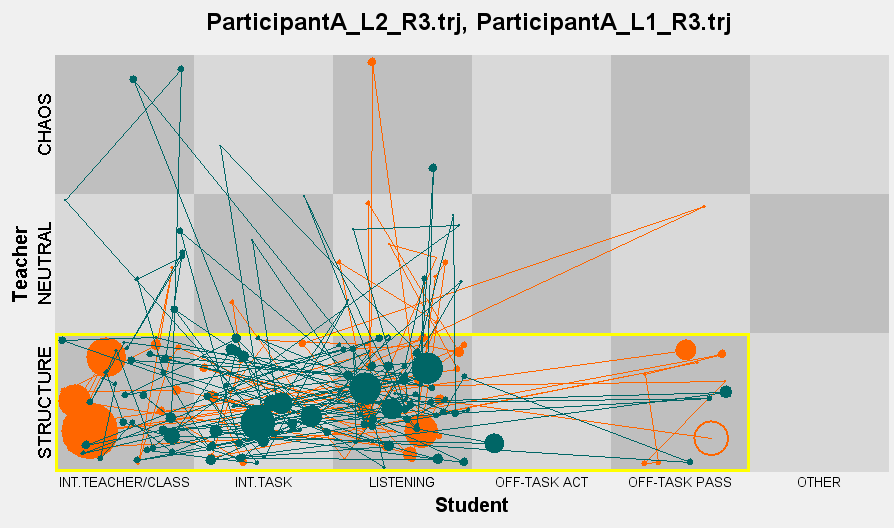


## Rafael and Cesar – Lessons 1 and 2


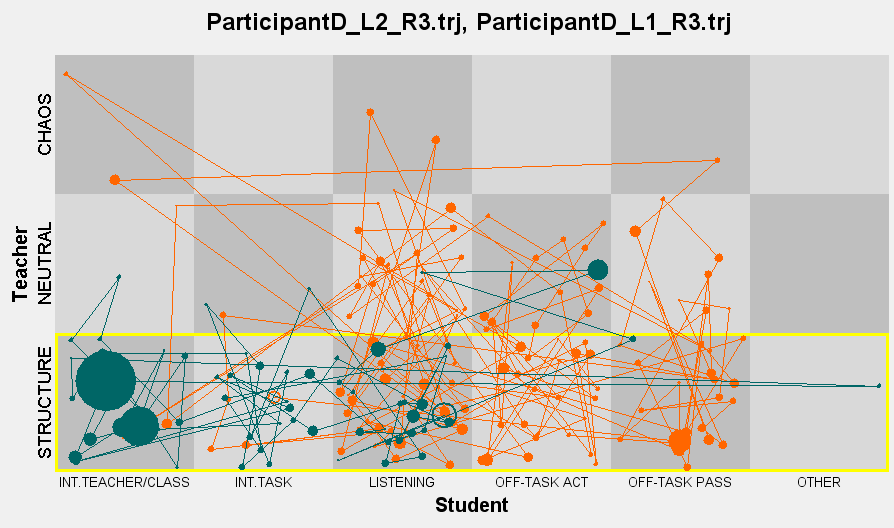


## Erick and Adrian – Lessons 1 and 2


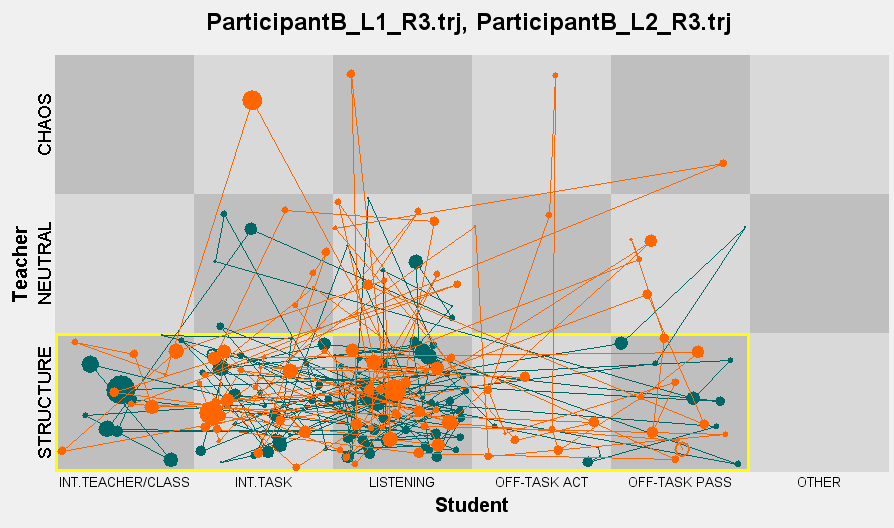


## Erick and Daniel – Lessons 1 and 2


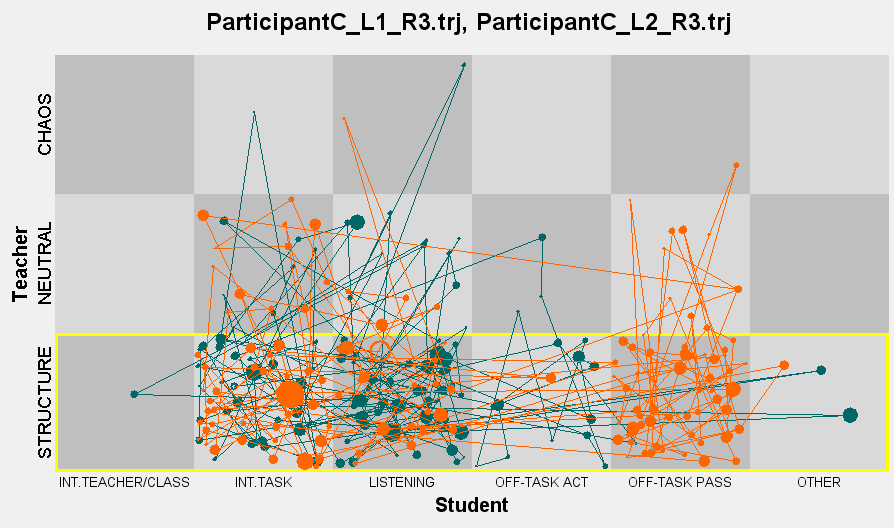


## Cindy and Alberto – Lessons 1 and 2


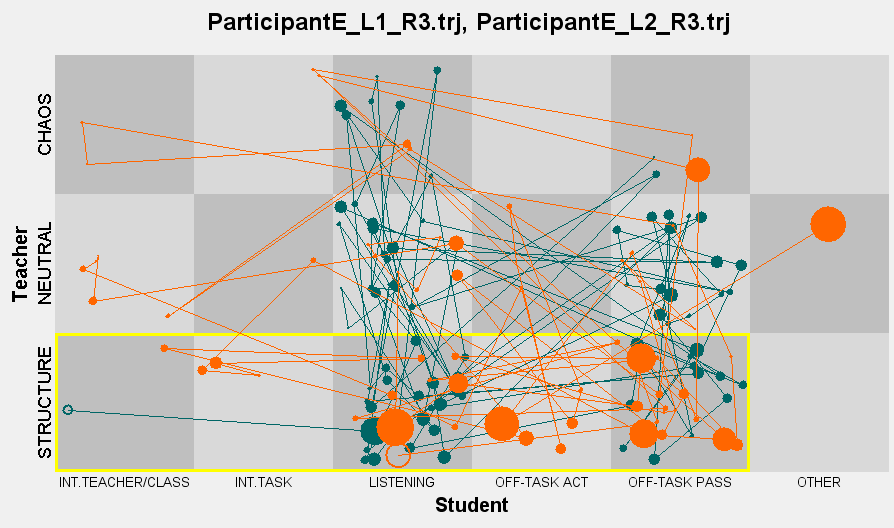


## Cindy and Jesus – Lessons 1 and 2


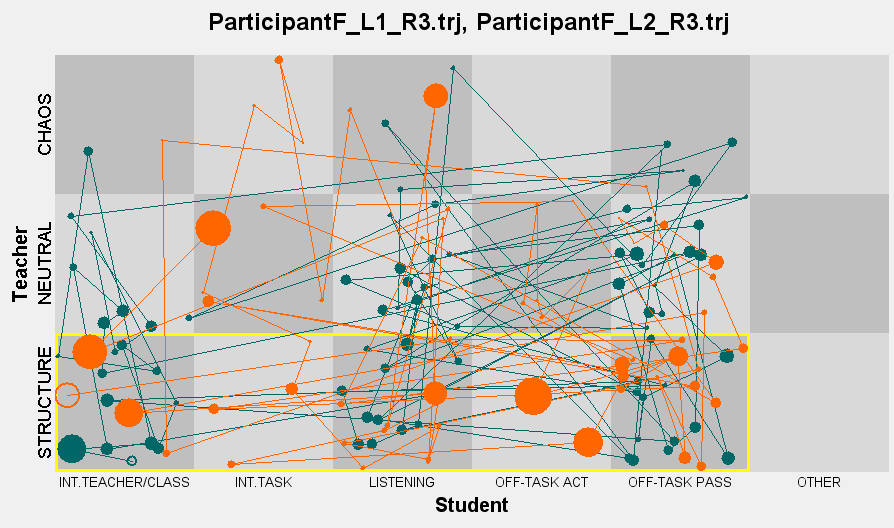


## Sofia and Sara – Lessons 1 and 2


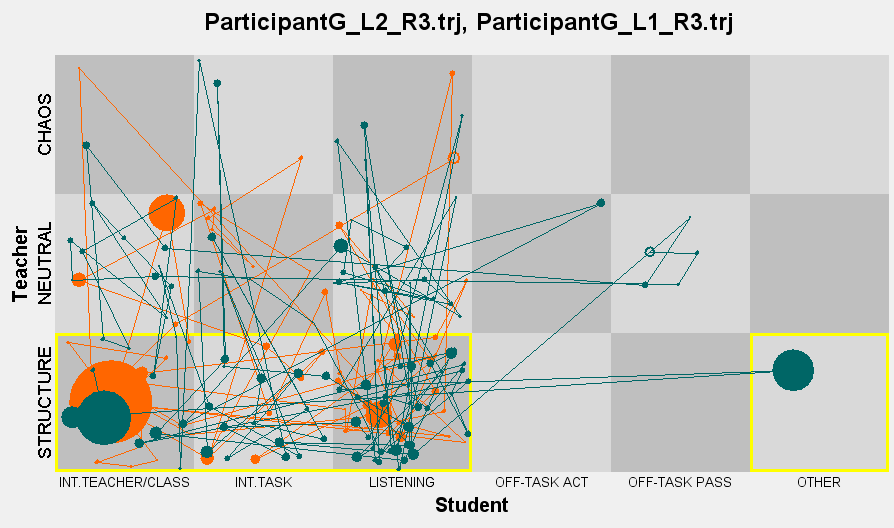


# State Space Grids for (teacher) involvement and student (dis)engagement

## Richard and David – Lessons 1 and 2


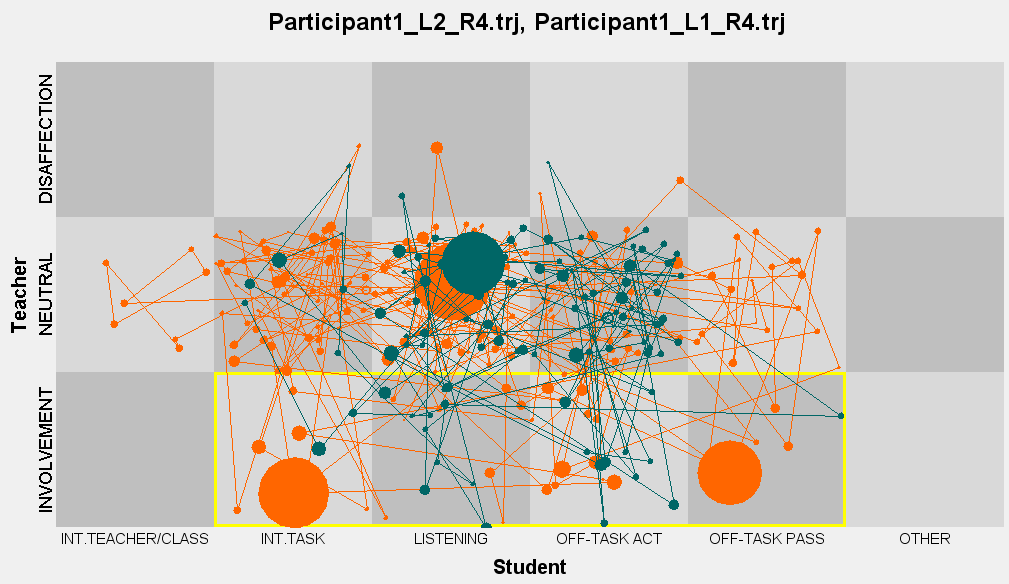


## Richard and Alan – Lessons 1 and 2


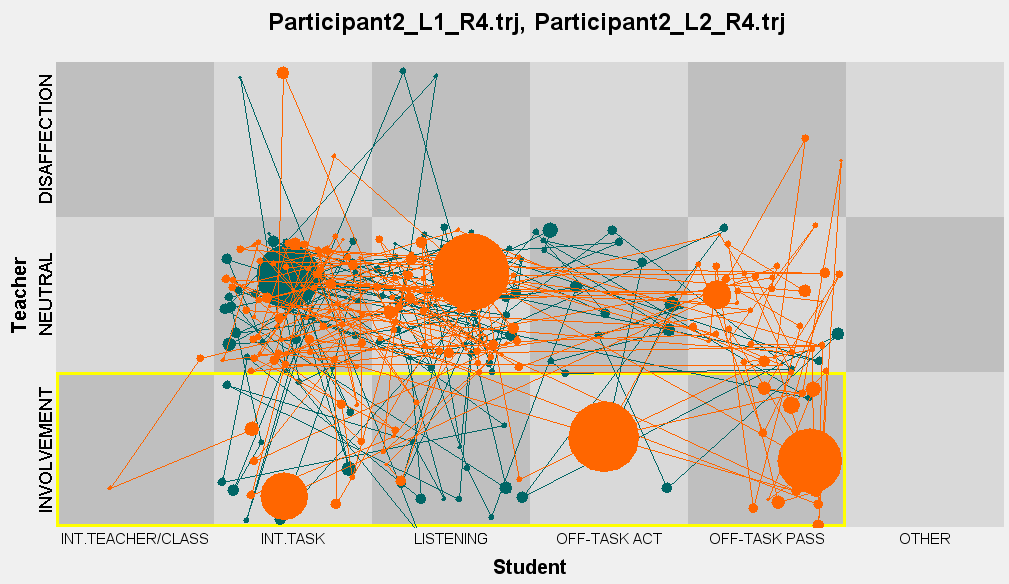


Note: The vertical axis illustrates the teacher states in the ‘involvement’ dimension: involvement, neutral and disaffection. The horizontal axis shows all student states, which can be ‘engaged’ (interacting with teacher/class, interacting with a task, listening), ‘disengaged’ (active, passive) or ‘other’. The region of interest (teacher displaying involvement and the student’s co-occurring behavior) is marked in yellow. The blue color represents Lesson 1, whereas the orange color denotes Lesson 2.

## Maria and Rachel – Lessons 1 and 2


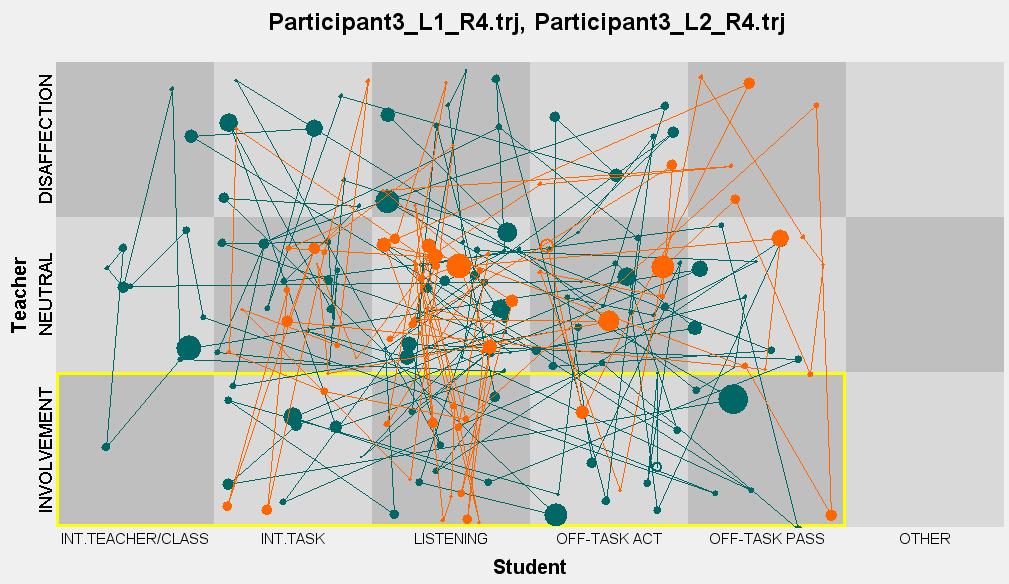


## Anne and Alex – Lessons 1 and 2


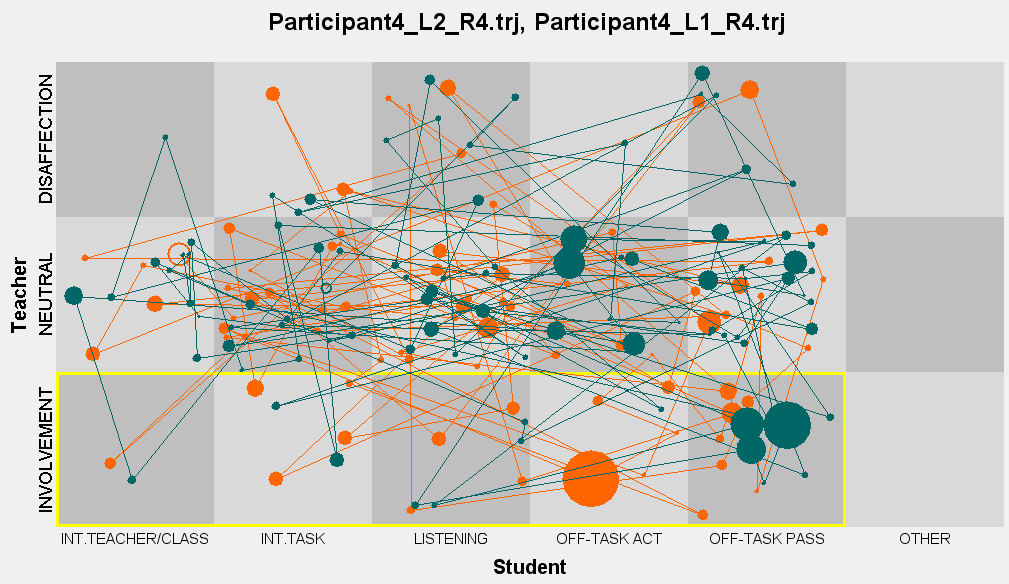


## Jack and Sandra – Lessons 1 and 2


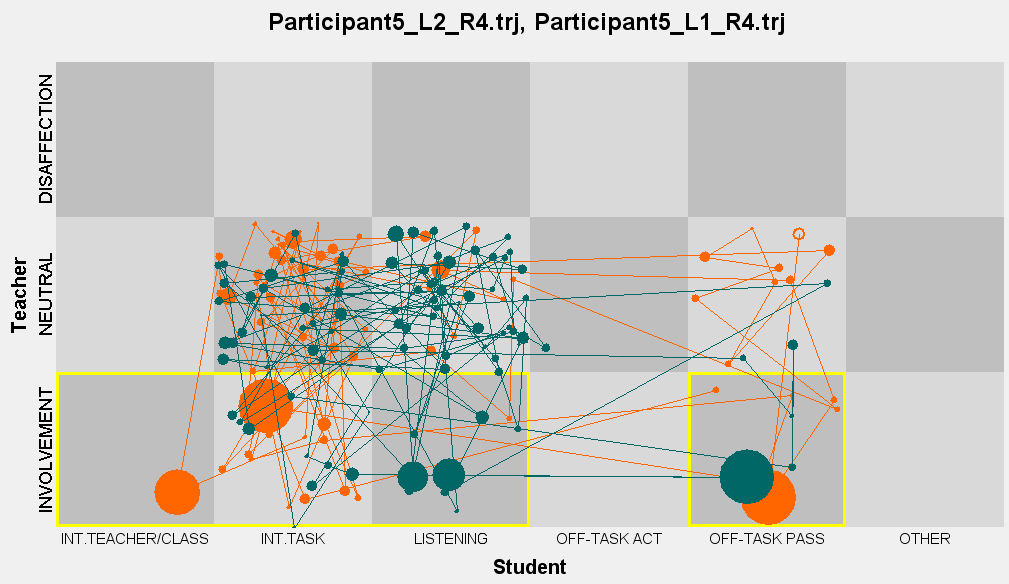


## Henry and Simon – Lessons 1 and 2


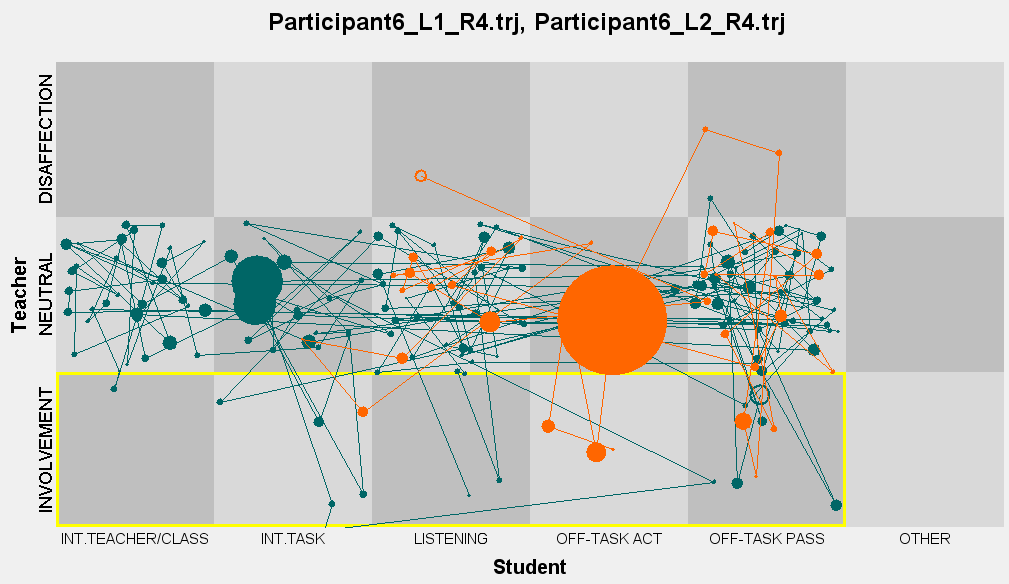


## Rafael and Sebastian – Lessons 1 and 2


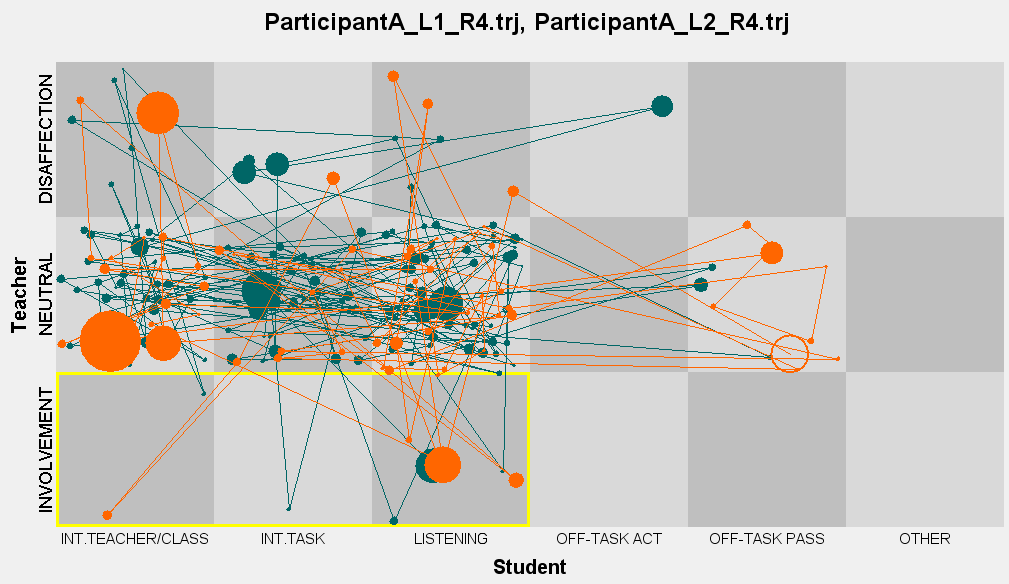


## Rafael and Cesar – Lessons 1 and 2


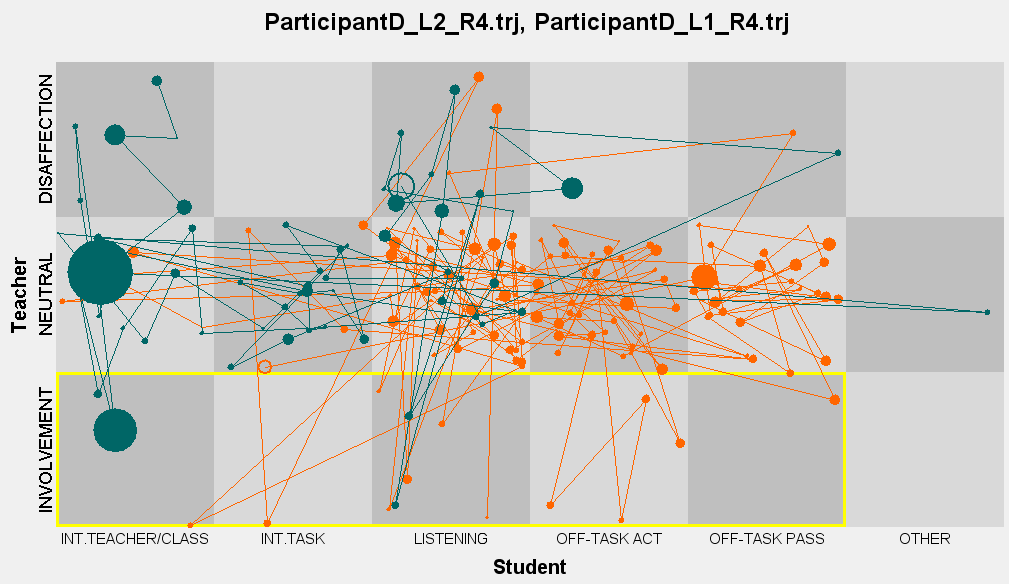


## Erick and Adrian – Lessons 1 and 2


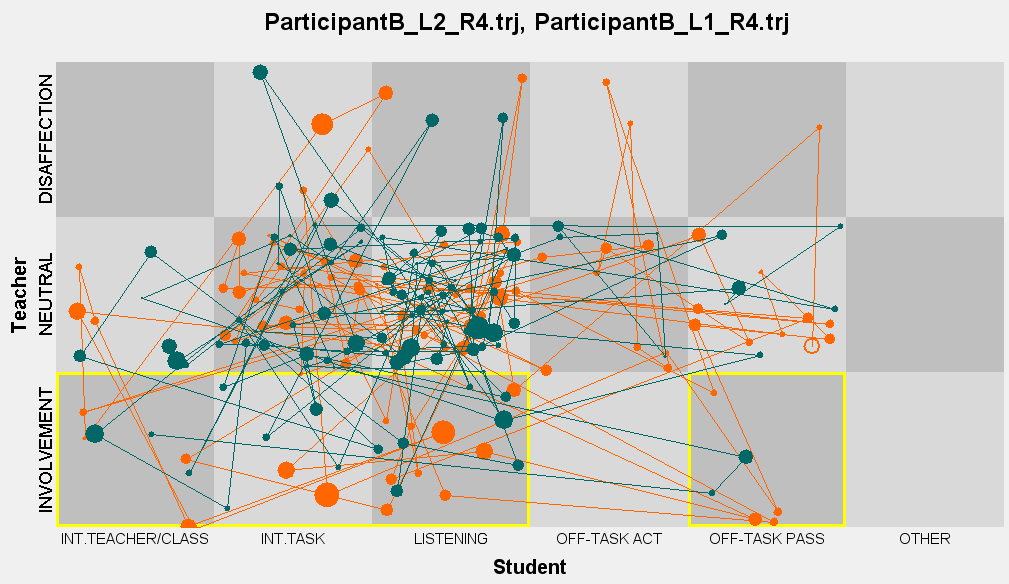


## Erick and Daniel – Lessons 1 and 2


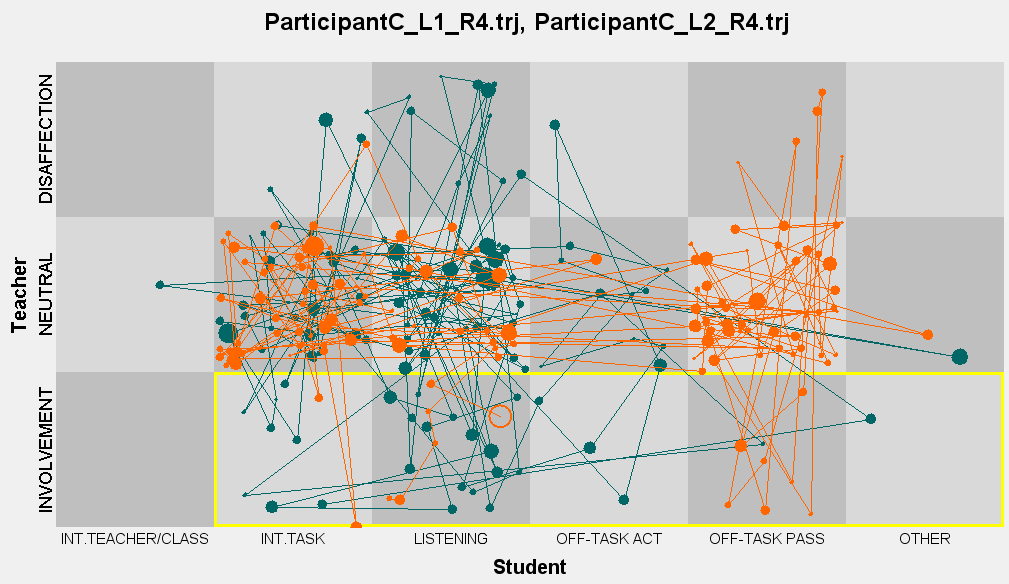


## Cindy and Alberto – Lessons 1 and 2


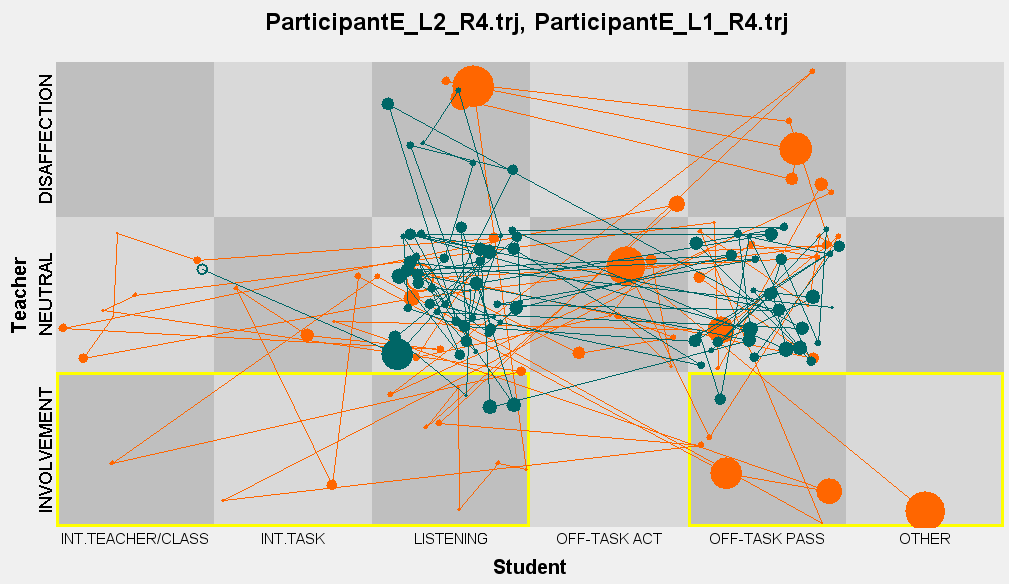


## Cindy and Jesus – Lessons 1 and 2


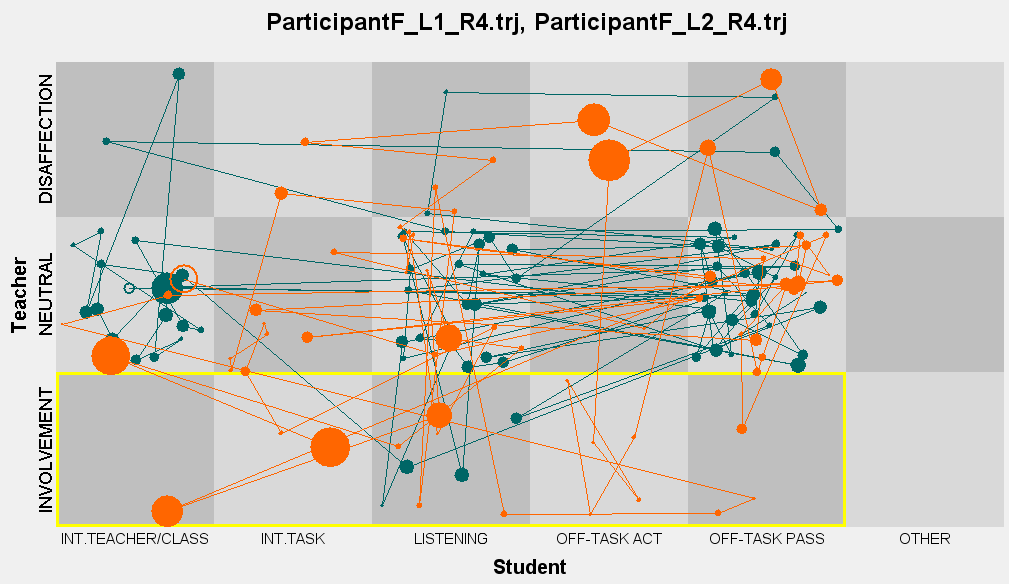


## Sofia and Sara – Lessons 1 and 2


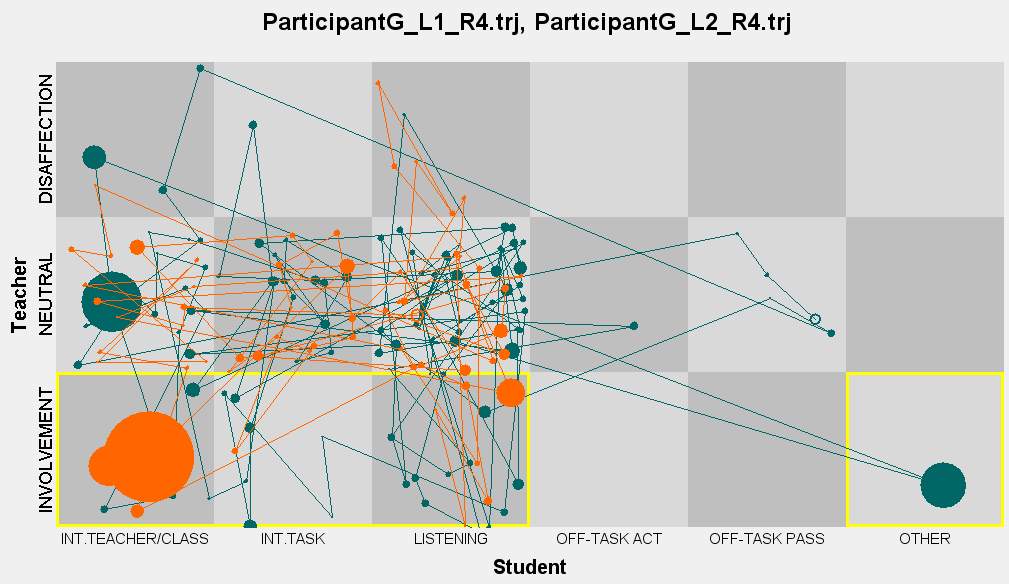

Supplement: Supplementary file 2 — Supplementary Material 2 [file 10803_2025_6723_MOESM2_ESM.docx]
